# Translucent Biocomposites from Hot-Pressed Wood Fibers and Poly(limonene acrylate)

**DOI:** 10.1021/acsami.5c07130

**Published:** 2025-07-21

**Authors:** Erfan Oliaei, Céline Montanari, Lengwan Li, Hui Chen, Peter Olsén, Lars Berglund

**Affiliations:** † Wallenberg Wood Science Center, Department of Fibre and Polymer Technology, 7655 KTH Royal Institute of Technology, Stockholm 10044, Sweden; ‡ State Key Laboratory of Organic-Inorganic Composites, College of Materials Science and Engineering, 47832 Beijing University of Chemical Technology, Beijing 100029, China; § Laboratory of Organic Electronics, 4566 Linköping University, Norrköping 60174, Sweden

**Keywords:** transparent biocomposites, pulp fibers, biobased thermoset, hot-pressed fibers, optical transmittance, eco-indicators

## Abstract

Translucent wood fiber composites offer new functions to stiff composites. Most “eco-friendly” thermoset resins are only partially biobased. Poly­(limonene acrylate), PLIMA, can be fully biobased and is combined with hot-pressed softwood fibers (WF) by liquid resin impregnation and curing. Fibers are random-in-plane or strongly oriented and have different lignin characteristics. Microstructure-mechanical property relationships are compared for hot-pressed WF networks and WF/PLIMA biocomposites from the same fibers. Stress transfer in WF/PLIMA biocomposites is enhanced with a modulus of up to 16.7 GPa and a tensile strength of up to 139 MPa, compared to transparent plastics like poly­(methyl methacrylate) (modulus ∼3 GPa, tensile strength ∼70 MPa). Optical transmittance is high, even at 35 vol % fiber content, suggesting translucent panels or lighting applications. Eco-indicators show that the PLIMA matrix accounts for ∼80% of biocomposite cumulative energy demand (CED, cradle to gate) of 60 MJ/kg, compared to ∼120 MJ/kg for glass fiber/thermoset composites.

## Graphical abstract

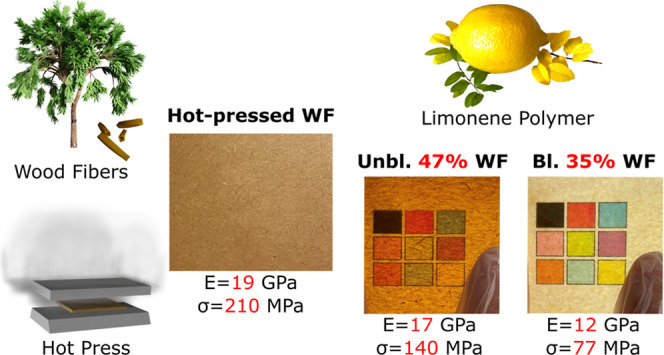

## Introduction

Wood has evolved to improve the mechanical function of trees while still providing tubular channels for the flow of water, molecules from photosynthesis, and liquid nutrients. Trees are larger than most other plants and can successfully compete for solar light because of their size. The tree trunks and branches are stiff and strong. The mechanical properties of the wood tracheid and fiber cell walls originate from oriented cellulose microfibrils embedded in a hydrated matrix of hemicelluloses and lignin.^1^ Although wood is a highly successful building material, the full potential of wood pulp fibers in engineering materials has not been realized. Wood offers little in terms of the fast processing of products with complex geometries, but wood pulp fibers can be used in molded polymer matrix composites as well as molded fibers. The technology of molded fibers is undergoing rapid development from traditional egg carton products.^2^ Thermoforming and hot molding in porous metal molds are used for the formation of more complex geometries. In addition, the material properties of molded fiber networks can be much improved by reducing the porosity.^2^

Wood pulp fibers are often produced from small-diameter trees or “sawmill waste”. The yield, in terms of planks and boards, from a sawmill is typically around 50%, and a large fraction of the “waste” is converted to wood chips for pulp mills. Chemical pulp fibers are predominantly produced by the kraft process. It is interesting that the cumulative energy demand (CED), the total energy used to produce a material, can be as low as 10 MJ/kg for wood pulp fibers,^3^ whereas polymers in the form of thermoplastic granules are typically in the range of 60–100 MJ/kg.^4^ A large pulp fiber application area is packaging materials. Because of technical–economical optimization, pulp fibers tend to be mechanically and chemically degraded in the final packaging board since interfiber bonding and mechanical properties of “paper sheets” (high-porosity fiber structures) can be improved by pulp fiber beating.^5^

In previous work, the hypothesis of improved mechanical properties of fiber materials made from mechanically and chemically undamaged wood fibers was tested. Mild chemical delignification was used in our lab to produce holocellulose fibers, where remaining cellulose and hemicelluloses were chemically well preserved compared with the native state^6^ and fibers showed little mechanical damage. Hot-pressed “molded fibers” with random-in-plane fiber orientation showed a modulus of 20 GPa and a tensile strength approaching 200 MPa at a porosity of around 21%.^6^ Typical low-cost thermoplastics have a modulus in the range of 1–4 GPa and strengths in the 20–60 MPa range. Arévalo and Peijs used flax fibers, which were disintegrated and hot-pressed to form materials of 17 GPa modulus and 120 MPa strength at 11% porosity.^7^ The main reasons for the superior properties of hot-pressed fibers reported by Yang et al.^6^ were high intrinsic mechanical properties of the fibers and strong interfiber adhesion. The holocellulose fibers were later combined with PMMA to produce polymer matrix composites with high mechanical properties and optical transmittance.^8^ In the context of sustainable development, however, the environmental impact of transparent holocellulose/PMMA composites should be reduced by considering more eco-friendly constituents.

The environmental impact of unbleached kraft pulp is lower compared to bleached grades. Bleached kraft pulp CED has been estimated at 14 MJ/kg,^2,3^ whereas unbleached kraft was reported as CED ≈ 9 MJ/kg.^2,3^ Reasons include higher yield of unbleached kraft pulp and reduced amount of pulping chemicals. We previously investigated hot-pressed unbleached kraft fiber materials with high mechanical properties, where residual lignin content was varied^9^ as well as fiber orientation distribution.^10^ Here, we will use a fully biobased polymer matrix^11^ to prepare biocomposites based on these fibers since moisture sensitivity and gas permeability are reduced for polymer biocomposites. In addition, more complex and much larger geometric shapes and structures can be manufactured. We will use the in situ synthesis of biobased polymer matrices within a reinforcing wood fiber network. This reduces energy demand compared with melt processing^12,13^ and should improve mechanical properties.

Acrylic acid can be efficiently obtained through biobased synthesis routes,^14^ and current industrial endeavors aim to commercialize biobased acrylic acid.^15^ While there are examples of methacrylate/acrylate originating from biobased sources in the literature,^16,17^ their chemical pathways often show limited environmental friendliness and relatively low efficiency. We therefore need new biobased acrylates with desirable material properties and simple synthesis pathways. We recently performed green synthesis of a novel limonene acrylate (LIMA) monomer from biobased resources.^18^ The LIMA monomer is suited for polymerization in wood fiber networks since free radical polymerization is insensitive to typical levels of residual pulp fiber moisture. This monomer has two distinct polymerization sites (an electron-poor acrylate group and an electron-rich alkene functionality) that upon radical propagation form a densely cross-linked polymer network and result in a transparent, amorphous thermoset. Poly­(limonene acrylate) (PLIMA) is fairly brittle but has a modulus of 2.2 GPa and a glass transition temperature as high as ≈130 °C. For transparent biocomposites, the refractive index (RI) of PLIMA is 1.52, whereas wood components have an RI of ≈ 1.54.^18,19^ This reasonably close match is encouraging because a larger mismatch in the refractive index increases light scattering.

A key to preparing transparent biocomposites from wood is to first remove light-absorbing chromophores, usually associated with lignin.^20–22^ In the next step, the wood reinforcement is impregnated by a polymer precursor to form a polymer matrix with a similar refractive index as the reinforcement, for high optical transmittance.^18,21^ Such composites offer unique opportunities for added optical functionalities.^23–25^ Here, fully biobased transparent wood fiber biocomposites are investigated since they offer design flexibility with tailored fiber orientation distribution. Established fiber composite processing methods (e.g., liquid molding) based on the present concept can be used for more complex geometrical shapes.

The objective is to investigate application-oriented processing and property aspects of hot-pressed industrial wood pulp fibers (bleached and unbleached) as candidate reinforcements in fully biobased polymer matrix biocomposites. This includes eco-indicators such as cumulative energy demand (CED). Technical aspects of biocomposite processing are discussed. The out-of-plane fiber orientation is characterized by wide-angle X-ray diffraction as well as the preferred fiber orientation in oriented molded fiber sheets. The effect of residual lignin (bleached and unbleached fibers) on the optical and mechanical properties was also investigated. Structure–property relationships for optical transmittance and mechanical properties of relevance for candidate applications are reported and compared for neat hot-pressed molded fibers and polymer matrix composites from the same fiber reinforcements. Biobased PLIMA polymerization is combined with hot-pressed wood fiber networks for translucent biocomposites with an up to ∼50% fiber volume fraction. Stress transfer effects from the polymer matrix are discussed and compared with those of hot-pressed neat fiber networks.

## Results and Discussion

### Materials Processing and Eco-indicators

An important objective was to prepare hot-pressed fiber networks and corresponding biocomposites with high fiber content (fiber volume fraction *V*_f_). This was successful using the process in Figure 1 (*V*_f_ ≈ 80% for fiber networks and *V*_f_ almost 50% for biocomposites). Industrial unbleached kraft pulp fibers (UBKP) from softwood with 14% lignin content were used as reinforcements in the form of fiber mats (paper sheets). UBKP has more favorable eco-indicators (lower cumulative energy demand and CO_2_ emissions) compared to bleached chemical pulp (BKP)^2,3,9^ due to higher yield and reduced use of chemicals. The UBKP fibers are mechanically undamaged and straight, with a low kink index, an average fiber length of 1.99 mm, and a width of 34.7 μm (Figures S1 and S2). Building upon previous work, where similar fibers showed good mechanical properties at porosities of 20–25% after hot-pressing,^9,10^ we here employed wet fiber sheets with either random-in-plane or oriented wood fibers (R-WF and O-WF), as shown in Figure 1a and detailed in the Experimental Section. Some O-WF sheets were chemically bleached (H_2_O_2_) to remove chromophores and reduce lignin content (8%), see Figure 1a. Limonene acrylate monomer can be synthesized from renewable resources^18^ and was used to impregnate hot-pressed wood fiber sheets, followed by free-radical polymerization into a thermoset matrix (Figure 1a). The limonene acrylate synthesis was designed to be eco-friendly, relying on biosourced reactants, minimizing hazardous chemical use, and reducing waste. The resulting biocomposites, as shown in Figure 1a, are transparent with a brown or slightly yellowish tint. The solvent exchange procedure described in the Experimental Section is expected to improve interfacial shear strength since it facilitates acrylate monomer diffusion into the porous fiber.^26^

**Figure 1:**
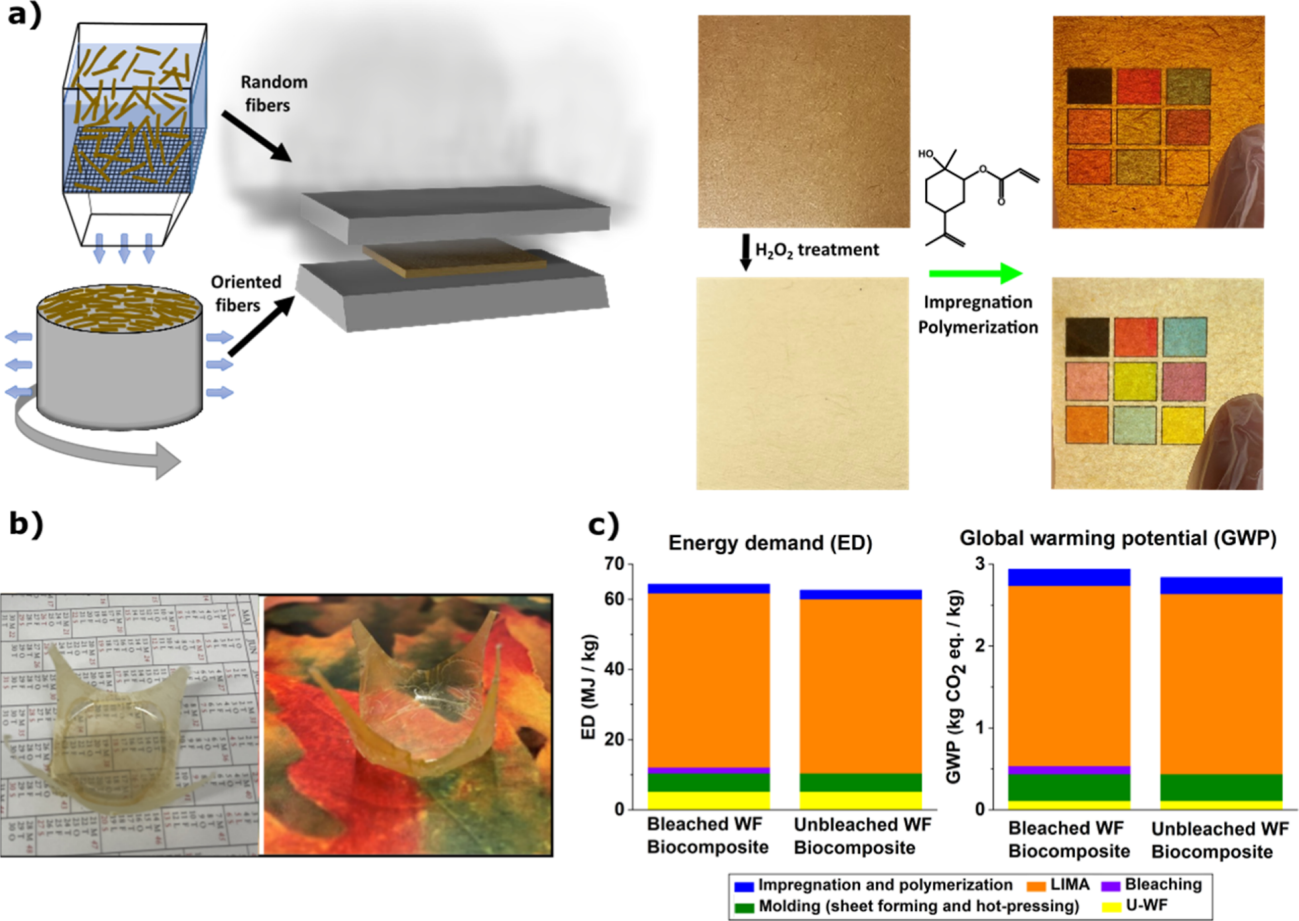
(a) The processing method for transparent biocomposites with random-in-plane or oriented wood fiber (WF) sheets. (b) WF/PLIMA biocomposites of more complex geometry prepared from “molded fiber” sheets. (c) Eco-indicators for energy demand and global warming potential for bleached and unbleached WF/PLIMA biocomposites including materials and processing steps (industrial).

The eco-friendly characteristics of biobased acrylic resins stem from their production routes. For example, recent techno-economic assessments^27^ discuss acrylic acid produced in a sugar cane biorefinery from renewable intermediates. In one pathway, lactic acid—obtained by fermenting carbohydrates from feedstocks such as corn, sugar beets, and cane sugar—is dehydrated to yield acrylic acid.^27,28^ Industrial efforts are underway for biobased acrylic acid.^15^ Similarly, limonene, a cyclic terpene extracted from citrus peel waste supplied from the juice industry,^29^ exemplifies the potential of biobased compounds. Limonene oxide and acrylic acid were reacted to synthesize limonene acrylate.^18^ Ethyl acetate was used to remove impurities or residual reactants. In an industrial process, ethyl acetate should be recycled to reduce waste. For green chemistry metrics, atom economy and reaction mass efficiency were evaluated.^30,31^ Limonene acrylate synthesis has 100% atom economy; however, the actual yield was 87%. The in situ polymerization of limonene acrylate inside molded wood fiber sheets has 100% atom economy and near-100% yield.

At an industrial scale, it would be desirable not to use acetone. Chemical fiber treatments, e.g., solvent-free esterification using biobased anhydrides (e.g., maleic, itaconic, or succinic), can be used for that purpose^32^ and also reduce moisture content. Figure 1b shows PLIMA/wood fiber biocomposites with a 3D structure.

The trees synthesize wood fibers, reducing dependency on fossil resources.^33^ Trees absorb CO_2_ from the atmosphere, and atoms become part of biopolymers, such as lignin and polysaccharides. We estimate cradle-to-gate cumulative energy demand (CED) (the energy required to produce 1 kg of material) and global warming potential (GWP) as eco-indicators for biocomposite material selection, similar to previous PLIMA analysis.^34^ For GWP, some components are directly derived from their energy demands using the average CO_2_ emission factors for Europe. This method is conservative (see Tables S3 and S4), since WF biocomposites produced in Sweden (where the power grid is dominated by hydroelectric and nuclear energy) emit only about 5% of the CO_2_ associated with the average European power grid. Further details are provided in the environmental impact assessment section of Supporting Information. A major advantage of LIMA is its fruit waste origin, although the upstream environmental impact needs improvement. LIMA synthesis from biosourced limonene oxide and bioacrylic acid yields a slightly lower CED of 49.6 MJ/kg compared to 54.4 MJ/kg for fossil-based sources (see Supporting Information). Limonene oxide, derived from citrus peel waste, avoids impacts from cultivation and harvesting. However, it has high water demand (148 kg wastewater/kg limonene^35^) and energy (11.3 MJ/kg). Bioacrylic acid, typically from lactic acid in sugar cane biorefineries, has a lower CED (22.8 MJ/kg) than its fossil-based counterpart (26.04 MJ/kg) and lowers GWP (see Supporting Information).

In lab-scale experiments, the energy demand is much higher than in industry (we calculated for the industrial scenario). For example, impregnation and polymerization of LIMA within a wood or wood fiber network in the lab requires ≈30 MJ/kg.^34^ In contrast, resin transfer molding results in much lower CED ≈ 2.6 MJ/kg^36^ (see Supporting Information).

The total CED is ∼60 MJ/kg, which is around 50% of typical values for glass fiber/thermoset composites^11^ and less than 60% of values for transparent or translucent poly­(methyl methacrylate) and polycarbonate sheets.^34^ The largest contribution to environmental impact is the production of LIMA (primarily due to its acrylic acid component), which accounts for roughly 80% of the total (Figure 1c). Even for biobased PLIMA, the energy demand is substantial^34^ compared to other contributions. In practical applications, PLIMA content should be minimized; alternative biobased monomers with lower energy demand are desirable, although criteria of high optical transmittance and a specific refractive index are limiting.

Free radical polymerization is suitable for the liquid molding of cellulose biocomposites. The use of biobased monomers in this process offers potential environmental benefits. Although there are vinyl monomer alternatives to LIMA (such as terpenes, methylstyrene, itaconic acid, and allylic cyclic carbonates),^37^ the majority of options in the literature tend to be acrylics, where the cradle-to-gate CED is unlikely to be much lower than for LIMA. In the present study, we are also restricted to transparent polymers with a refractive index similar to PLIMA (and the wood substrate).

### Composition and Microstructure of Fibers, Hot-Pressed Fibers, and Polymer Matrix Biocomposites

The molecular-scale mixture of lignin/hemicellulose biopolymers in the WF appears to help interfiber adhesion in hot-pressed fiber sheets.^9^ The unbleached WF sheets contained 14% residual lignin and 19% hemicellulose, while the bleached WF sheets contained 8% residual lignin and 10% hemicellulose. Because of the nature of the bleaching process, the difference in lignin content at fiber surfaces might be even larger, which may influence interfiber adhesion during hot-pressing.

FTIR spectra in Figure 2a show the effects of bleaching on the hot-pressed WF sheets. Unbleached fibers show higher intensity of nonaromatic C=C peaks, which means that those fibers may react with limonene acrylate during polymerization. For bleached fibers, the intensity of peaks corresponding to carbonyl groups in lignin and hemicellulose is reduced (1740, 1711, and 1640 cm^–1^).^38,39^ The C=C stretching peak of aromatic rings in lignin (1509 cm^–1^) and other related peaks are also reduced (e.g., C=C stretching at 1600–1640 cm^–1^, related to aromatic rings and nonaromatic bonds).^40,41^ In addition, the C–H stretching band (2930 cm^–1^) and O–H stretching band (3430 cm^–1^) showed reduced absorption intensity^42^ with bleaching. The reduction in C=C peaks relates to the reduced content of lignin and chromophores.

**Figure 2:**
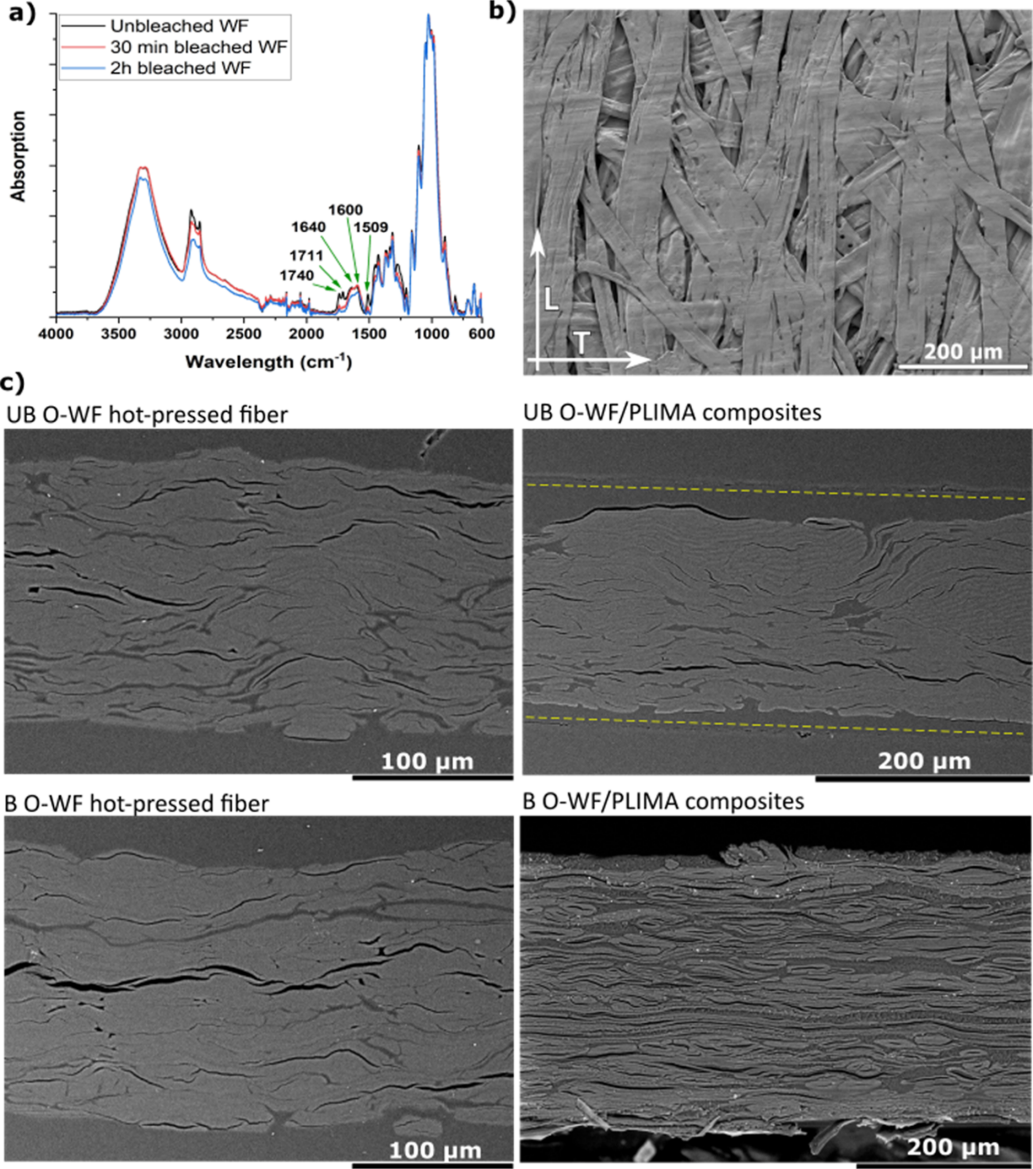
(a) FTIR spectra of unbleached wood fiber sheets and 30 min bleached and 2 h bleached wood fiber sheets. (b) SEM images from the surfaces of O-WF sheets. (c) Cross sections of unbleached wood fiber sheet (UB O-WF, *V*_f_ = 80%), bleached wood fiber sheet (B O-WF, *V*_f_ = 76%), wood fiber poly­(limonene acrylate) biocomposite (UB WF/PLIMA, *V*_f_ = 47%), and poly­(limonene acrylate) bleached wood fiber biocomposite (B WF/PLIMA, *V*_f_ = 35%). Cross sections of wood fiber sheets are prepared by epoxy embedding and polishing.

SEM images of cross sections are presented in Figure 2. Again, hot-pressed fibers reached up to *V*_f_ = 80%, while biocomposites reached almost *V*_f_ = 50%, which is difficult to achieve without hot-pressing. The surface of a hot-pressed, unbleached, and oriented O-WF sheet is presented in Figure 2b. The preferred orientation is apparent, as is the undamaged nature of the high-aspect-ratio fibers (length/diameter ≈2 mm/30 μm ≈ 67), although the fibers are collapsed with ribbon-like cross sections. The nature of the porosity in the sheets is observable from this micrograph; full monomer impregnation of dense sheets (porosity 20%, see Figure S3) is challenging, although slightly easier for randomly oriented fiber sheets of 24% porosity, see Figure 2c. Cross-sectional micrographs of the O-WF fiber sheets (Figure 2c, main fiber direction out of the cross-sectional plane) reveal the nature of voids in this plane in the form of interfiber gaps and collapsed fiber lumen voids (note that these fiber sheets were also impregnated with resin in order to facilitate specimen preparation for microscopy).

The cross sections of oriented O-WF/PLIMA biocomposites are in the right column of Figure 2c. It is apparent that the process was successful in providing biocomposites with high fiber content (*V*_f_ = 47% and 35%). Unbleached fiber composites have a thickness of 240 μm, while bleached ones are 300 μm; see Table S1. Bleached composites are a bit thicker due to reduced surface polymer layer and increased fiber network porosity, which increases matrix uptake, consistent with SEM and preimpregnation WF sheet thickness data (∼190 μm vs ∼180 μm, Table S1). The thickness data are summarized in Table S1. The UB O-WF/PLIMA biocomposite has a polymer surface layer of ∼25 μm. In WF/PLIMA biocomposites, most of the interfiber space is filled with the polymer matrix, although some thin vertically elongated voids or gaps are apparent. Many voids are lumen spaces at the center of the hollow fibers. Although impregnation is successful in producing high *V*_f_ biocomposites, the dense nature of hot-pressed WF sheets makes the preparation of void-free composites challenging.

Hermans’ orientation parameter (*f*) was determined for both random-in-plane and oriented fiber sheets and composites using WAXD. This technique measures the orientation of cellulose crystals embedded in fibrils, which are aligned at specific angles relative to the fiber axis (Figure S11). The Hermans’ orientation parameter, derived from the diffraction pattern, quantifies the degree of this alignment (see Supporting Information). The out-of-plane orientation measured from the specimen edge (a measure of how planar the fibers are *f*_L_ or *f*_W_ in Supporting Information) was estimated, and the orientation state of the O-WF fiber sheets was measured with the X-ray beam perpendicular to the major plane of the specimen (*f*_P_). The high values of *f*_L/W_ (0.69–0.73) primarily indicate fibril alignment, reflecting limited out-of-plane orientation or waviness of the wood fibers during the sheet forming and drying process, which is crucial for maintaining a high in-plane modulus. Hermans’ parameter, *f*_P_, for O-WF is high (0.69–0.70) compared to previous data for oriented holocellulose fiber sheets (0.56)^8^ but similar to the *f*_P_ of unbleached fibers of O-WF we have analyzed previously (0.71).^10^ This high value of *f*_P_ could be attributed to the stiffness and straightness of unbleached fibers aligning well with the drum rotation. The *f*_P_ of unbleached O-WF is 0.69, corresponding to an average off-axis crystal angle of <φ> = ± 27°. The weight-average off-axis angle of the fibers from SEM images is 26.1°. The agreement between the two measurements is encouraging. Further discussion of the relationship between cellulose fibril orientation and wood fiber orientation can be found in Supporting Information.

The extent of the out-of-plane fiber orientation is also important for modulus data. The cross-sectional Hermans’ orientation parameters are high in both O-WF and R-WF molded fibers and corresponding biocomposites (*f* = 0.69–0.73), which means limited out-of-plane misalignment. The in-plane modulus is strongly reduced with an increasing out-of-plane orientation angle. Further data are provided in Supporting Information.

Bleaching of the unbleached O-WF resulted in increased crystallinity, due to removal of noncellulosic amorphous components (lignin and hemicelluloses). This measure of crystallinity is not cellulose crystallinity but an estimate of “fiber” crystallinity. The crystallinity measured in the lengthwise direction increased from 20% to 22% due to bleaching, although the intrinsic cellulose crystallinity may not have changed. Cellulose crystal size, however, can increase in bleached molded fibers due to cellulose microfibril aggregation.^43–45^ Biocomposites showed even larger cellulose crystallites, possibly related to annealing effects from an elevated temperature during polymerization.

Moisture sensitivity was also evaluated by conditioning wood fiber sheets, neat PLIMA, and biocomposite samples at 99% relative humidity (RH) and 23 °C, with 50% RH and 23 °C as the baseline (details in the Supporting Information). Moisture absorption (weight gain) and dimensional stability (thickness change) were measured over 16 days. Biocomposites with PLIMA showed significantly lower moisture uptake (∼3–4%) compared to pure wood fiber sheets (∼6–8%) due to the protective effect of the hydrophobic polymer matrix. PLIMA showed minimal moisture uptake of ∼1.2% weight gain. Thickness swelling was generally low (less than ∼5%) and more stable in biocomposites than in wood fiber sheets, with PLIMA showing the least change.

### Optical Properties of Polymer Matrix WF/PLIMA Biocomposites

A biocomposite with high optical transmittance requires low absorption (no chromophores) and a polymer with a refractive index (RI) close to the reinforcing fiber.^20^ For the bleached wood fibers, the RI is 1.53–1.54,^19^ while PLIMA has an RI of 1.52,^18^ which is a closer match compared with PMMA (RI = 1.49). Optical transmittance is measured using an integrating sphere.^46^ Haze is defined as the ratio of transmitted light scattered at an angle of more than 2.5° from the incident direction of the beam to the total transmitted light. Haze data depend on the sample thickness and geometry of the integrating sphere, especially for high-scattering materials. The optical properties of wood fiber biocomposites, including total transmittance, haze, absorption, and attenuation coefficient, depend on fiber volume fraction, fiber orientation distribution, defects at fiber–polymer interface, and lignin content.^47^ The pure PLIMA film of 1.2 mm thickness showed a total transmittance of 92% at 550 nm and a low haze of 2.5% (Figure S4 and Table S1). Figure 3a,b presents photographs of WF/PLIMA biocomposite samples made from unbleached and (2 h) bleached fiber sheets, with 47% and 35% fiber volume fractions (*V*_f_), respectively. The sample with unbleached fibers (Figure 3a) shows a brownish tint due to lignin, which absorbs light in the blue light range.^25^

**Figure 3:**
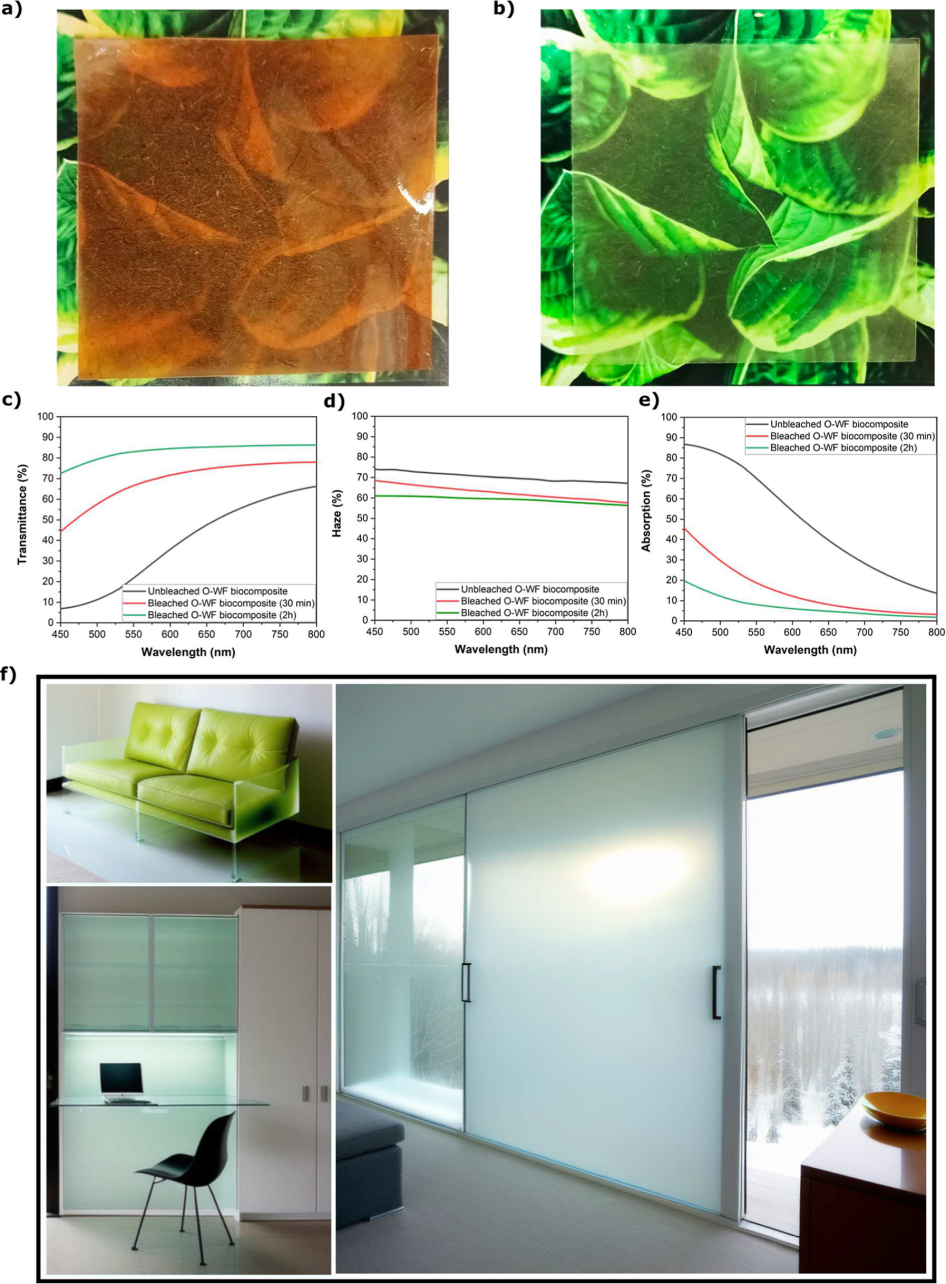
PLIMA biocomposites based on (a) unbleached WF sheets (*V*_f_ 47%) and (b) 2 h bleached WF sheets (*V*_f_ 35%). (c) Transmittance, (d) haze, and (e) absorption of WF/PLIMA biocomposites based on an unbleached WF sheet and WF sheets with 30 min and 2 h of bleaching. Biocomposites thicknesses: UB O-WF/PLIMA 240 μm and B O-WF/PLIMA 300 μm. (f) Potential applications: furniture, light diffuser, and translucent “windows”. Images were created using AI-assisted graphic generation based on author-described vision.

Figure 3c shows that the optical transmittance of the B WF/PLIMA biocomposite bleached for 2 h (0.3 mm thick) reaches 83% at 550 nm, significantly higher than for the other two materials (see Table S1 for details). Additionally, Figure 3d shows that the haze is lower for this biocomposite. Bleaching has a favorable effect on transmittance; however, the WF sheet remains yellowish even after 2 h, indicating that absorption due to chromophores still reduces transmittance of the B WF/PLIMA biocomposite. As a result, biocomposites produced under these conditions show visible coloration.

The transmittance as a function of wavelength in Figure 3c shows that lignin removal decreases the absorption in shorter wavelengths. There is also higher transmittance of the B WF/PLIMA biocomposites at the highest wavelength tested (800 nm), where there are fewer effects from lignin absorption. The reason may be a better matching of the refractive index with PLIMA. Additionally, a more porous structure of the wood fiber cell walls from bleaching may facilitate polymer impregnation, for increased transmittance and reduced haze. For potential applications of biocomposites made by liquid molding, examples could be translucent panels for furniture and decorative purposes (Figure 3f). At current thickness ∼0.3 mm, the composites lack sufficient mechanical stability and thermal insulation for standard window applications, which typically require 3–6 mm thick glass. They are interesting for nonstructural uses like light diffusers or decorative translucent panels in furniture. Increased thickness via lamination would enable semistructural applications, such as furniture or cupboards, although light scattering will increase.

Compared to the literature, Yano et al. developed a 50 μm-thick acetylated fiber sheet impregnated with acrylic resin, achieving a total transmittance of 88%.^48^ However, the composite’s cellulose content was not so high (28 wt %, *V*_f_ ∼ 23%). Zha et al.^49^ produced holocellulose WF/acrylic biocomposites from holocellulose WF sheets, with a fiber volume fraction (*V*_f_) of 49%. This sample attained an optical transmittance of ≈72% at a thickness of 60 μm. “Transparent wood” composites often achieve high transmittance levels (>80%) at thicknesses of approximately 1 mm.^18,21,25^ However, the cellulose content of “transparent wood” is often low, ranging from 5 v % to 25 v %.^18,21,47^

The attenuation coefficient is a robust, intrinsic parameter for comparing optical transmittance of materials since it quantifies the fraction of light lost per unit thickness, independent of sample dimensions.^50,51^ However, its accurate determination requires measurements for several thicknesses.^52^ Here, we collected data for different transparent WF composites (see Supporting Information). High values indicate low transmittance. Literature shows WF acrylic with *V*_f_ ∼ 23% and WF *V*_f_ ∼ 49% with coefficients of 7.6 cm^–1^ and 20.8 cm^–1^, respectively.^48,49^ For the present bleached B WF/PLIMA (*V*_f_ = 35%), the calculated coefficient was 2.1 cm^–1^, which is encouraging for high wood content and favorable mechanical properties (discussed in the next section). Transparent wood materials with lower wood content, such as TW PMMA (*V*_f_ 12%) and TW PLIMA (*V*_f_ 12%), show lower attenuation coefficients of 1.7 cm^–1^ and 0.4 cm^–1^, respectively, but have low mechanical properties. The mechanism for the effects of wood content on attenuation coefficient is discussed by Chen et al.^52^ The attenuation coefficient depends not only on light absorption but also on light scattering effects (anisotropic diffusion coefficients).

Table 1 shows the variation in the attenuation coefficient with composition, highlighting the influence of both absorption and scattering. A lower attenuation coefficient correlates with reduced wood fiber volume fraction and better refractive index matching, leading to less scattering. Literature data on transparent wood and wood fiber composites, summarized in Table 1, represent combined effects from absorption (e.g., lignin content) and scattering (e.g., fiber content and refractive index mismatch) on the attenuation coefficient and transmittance. Understanding the interplay between absorption, scattering, and material anisotropy is crucial for applications such as transparent panels, where a minimized attenuation coefficient enhances transmittance and may reduce haze.

**Table 1:** Fiber Volume Fraction and Attenuation Coefficients of Transparent Wood and Wood Fiber Composites

material	fiber volume fraction (*V*_f_) (%)	RI mismatch (reinforcement/polymer) (ΔRI)	attenuation coefficient (cm^–1^)
WF acrylic^48^	∼23	**0.03** (1.52/∼1.49)	7.6
WF acrylic^49^	∼49	**0.03** (1.52/1.49)	20.8
B WF/PLIMA (present study)	35	**0.01** (1.53/1.52)	2.1
TW PMMA^52^	12	**0.04** (1.53/1.49)	1.7
TW PLIMA^18^	12	**0.01** (1.53/1.52)	0.4

Scaling to thicker samples is interesting for load-bearing applications, although optical transmittance will decrease exponentially with increased thickness, primarily due to increased scattering when the number of cellulose–polymer interfaces is increased. Mitigating this for a polymer with a given refractive index requires a reduction in optical manufacturing defects as well as a more homogeneous distribution of cellulose and polymer matrix at a very small scale.

### Mechanical Properties of Neat, Hot-Pressed Fibers and Polymer Matrix Biocomposites

The stress–strain curves for neat PLIMA, random (R-WF, only unbleached) and longitudinally oriented wood fiber sheets (L-WF), and their biocomposites are shown in Figure 3a,b (data are summarized in Table 2). The fiber volume fractions *V*_f_ of the unbleached random composites (UB R-WF/PLIMA) and oriented composites (UB O-WF/PLIMA) are 51% and 47%, respectively. The *V*_f_ of bleached oriented composites (B O-WF/PLIMA) is substantially lower, at 35%. The bleached R-WF network could not be tested since it disintegrated from poor interfiber adhesion and thus no bleached R-WF composites either.

**Table 2:** Mechanical and Physical Properties of the WF Fiber Network Sheets and WF/PLIMA Biocomposites^a^

sample	material	porosity (%)	*V*_f_ (%)^b^	modulus *E* (GPa)	ultimate strength σ_U_ (MPa)	strain at break ε_u_ (%)	effective fiber modulus *E*_f_ (GPa)	effective fiber strength σ_f_ (MPa)
PLIMA	neat polymer	0	0	2.2 (0.4)	15 (7)	0.7 (0.3)		
UB R-WF	fiber network	24	76	10.8 (0.4)	118 (7)	2.3 (0.3)	38	
UB R-WF/PLIMA	biocomposite	<5	51	11.7 (1.1)	106 (5)	1.7 (0.3)	52	
UB L-WF	fiber network	20	80	18.7 (0.5)	210 (6)	2.1 (0.1)	39	438
B L-WF	fiber network	24	76	13.0 (1.5)	67 (5)	0.9 (0.2)	29	139
UB L-WF/PLIMA	biocomposite	<5	47	16.7 (2.5)	139 (9)	1.3 (0.2)	55	464
B L-WF/PLIMA	biocomposite	<5	35	11.6 (1.3)	77 (5)	0.9 (0.1)	48	320
UB T-WF	fiber network	20	80	6.0 (0.6)	65 (4)	1.8 (0.3)		
B T-WF	fiber network	24	76	3.0 (0.3)	23 (1)	3.0 (0.3)		
UB T-WF/PLIMA	biocomposite	<5	47	6.7 (1.0)	41 (2)	0.6 (0.3)		
B T-WF/PLIMA	biocomposite	<5	35	4.1 (0.1)	28 (1.2)	1.2 (0.1)		

a B WF refers to the 2 h bleached WF sheet. Assuming ρ_s_ 1.5 g/cm^3^ as the theoretical density of lignocellulose, the porosity of WF sheets was calculated. The measured density of neat PLIMA was 1.13 g/cm^3^. The WF sheet density is ρ*, and *V*_f_ is calculated as ρ*/ρ_s_.

b For biocomposites, the reported fiber volume fraction excludes polymer porosity. For the calculation of effective fiber properties, see Supporting Information.

In Figure 4a, the unbleached R-WF/PLIMA composite shows a tensile strength σ_U_ = 106 MPa and an elastic modulus of *E* = 11.7 GPa, comparable to the properties of the neat R-WF sheet, despite having a lower volume fraction of fibers (51% vs 76% for the neat, hot-pressed R-WF sheet). The reason is that the PLIMA matrix enhances stress transfer to fibers by filling interfiber gaps, creating a continuous phase that also distributes stress more uniformly and reduces stress concentrations, unlike the fiber network with a 24% porosity. Note that neat PLIMA is a brittle thermoset with an elastic modulus of *E* ≈ 2.2 GPa and a tensile strength of σ_U_ ≈ 15 MPa.

**Figure 4:**
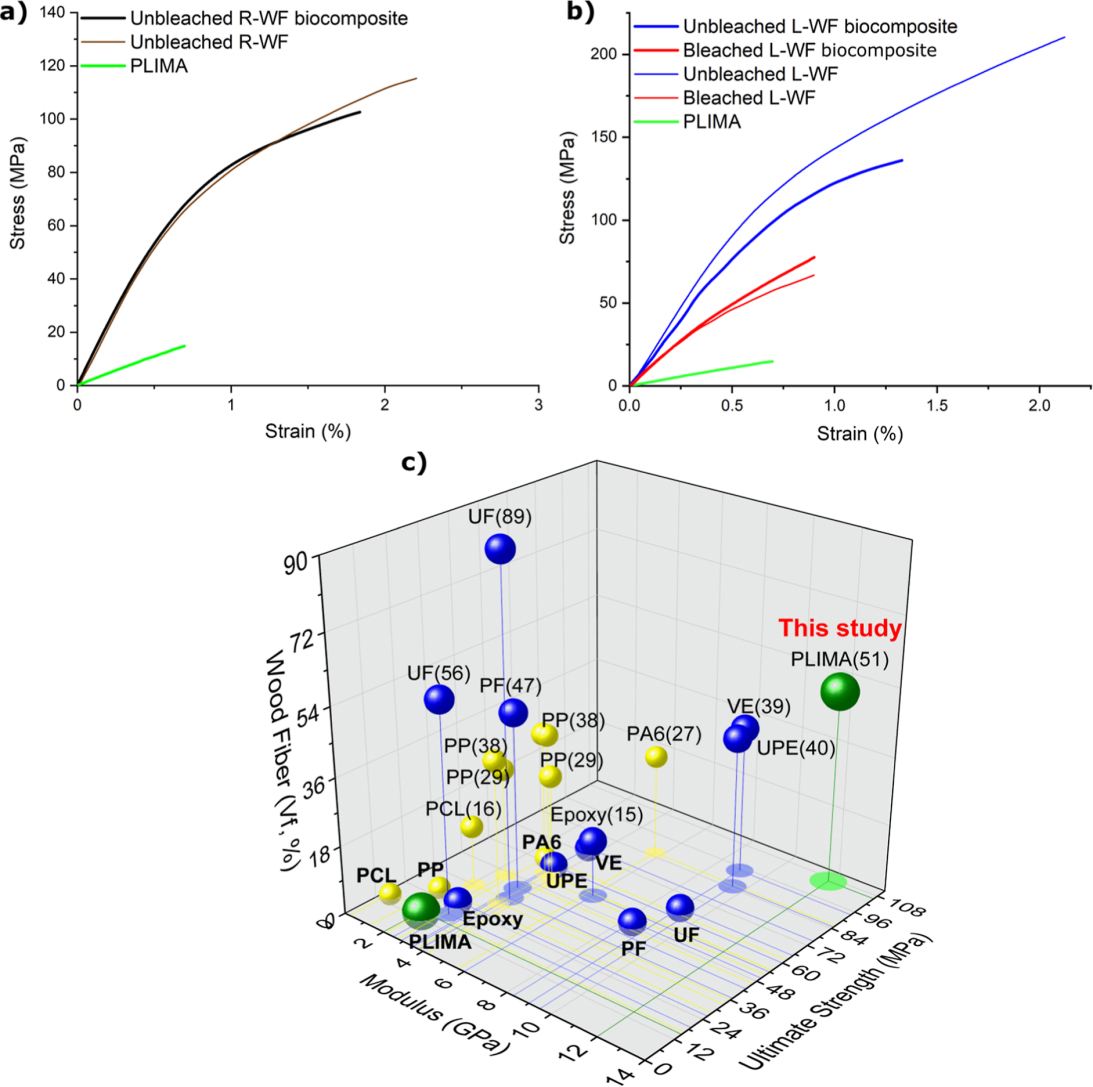
Representative tensile curves of (a) random-in-plane wood fiber biocomposites (bleached and unbleached, B and UB, respectively) and (b) oriented wood fiber biocomposites. See Figure S6 and Table 2 for data transverse direction. (c) Comparison of elastic modulus and tensile strength of wood fiber (WF) biocomposites. All composites in (c) have random-in-plane fiber reinforcement, with thermoplastic composites in yellow and thermoset composites in blue. Composites are named after the matrix polymer and fiber weight fraction. Literature data includes vinyl ester (VE),^53,54^ urea-formaldehyde (UF),^55–57^ unsaturated polyester (UPE),^53,57^ phenol-formaldehyde (PF),^55,57^ epoxy,^58,59^ polyamide 6 (PA6),^60,61^ polylactic acid (PLA),^59^ polypropylene (PP),^62–65^ and polycaprolactone (PCL)^66^ composites. See Figure S7 for 2D literature data representation.

The oriented hot-pressed unbleached oriented wood fiber (UB O-WF) sheets, with a solid volume fraction (*V*_f_) of 80%, showed an ultimate strength (σ_U_) of 210 MPa and a modulus (*E*) of 18.7 GPa in the longitudinal direction. In contrast, the bleached hot-pressed oriented wood fiber (O-WF) sheet, with a slightly lower *V*_f_ of 76%, showed significantly reduced mechanical properties: σ_U_ = 67 MPa and *E* = 13 GPa in the longitudinal direction (see Figure 4b and Table 2). For neat WF sheets in Figure 4b, the porosities are comparable (20% for UB O-WF and 24% for bleached O-WF). The decrease in both strength and modulus in the bleached neat fiber networks suggests that bleaching reduces interfiber bonding. The lower modulus in the bleached sheet is in strong support of this interpretation, which could also explain the lower strength due to unfavorable failure mechanisms.

The oriented UB O-WF/PLIMA biocomposite, with *E* = 16.7 GPa and σ_U_ ≈ 140 MPa but lower fiber content (*V*_f_ ≈ 47%), is weaker than neat hot-pressed UB O-WF fiber sheets (*V*_f_ ≈ 80%) in the longitudinal direction, Figure 4b. For oriented fibers, interfiber adhesion is less important, and the higher *V*_f_ is likely to explain the higher strength of UB O-WF fiber sheets. For oriented composites, bleached B O-WF/PLIMA (red curve) shows lower *E* and σ_U_ values than UB O-WF/PLIMA (blue curve) in the longitudinal direction, as shown in Figure 3 and Table 2. One reason is lower fiber content (*V*_f_ = 35% vs *V*_f_ = 47%), but this only explains the modulus difference (effective fiber moduli *E*_f_ are similar, see Table 2). The effective fiber strength σ_f_ is lower for bleached fiber composites (320 vs 464 MPa, Table 2), and one may speculate that this type of bleaching reduces σ_f_.

There is a similarity between the stress–strain curves of the composites and their corresponding hot-pressed fiber sheets (red curves are similar and blue curves are similar), suggesting that fiber network behavior influences composites’ behavior. There are probably many interfiber bonds remaining from hot-pressing, where no polymer matrix is present as a separating layer between individual fibers.

For oriented fibers and biocomposites, the weakest mechanical properties transverse to the fiber orientation direction are of interest. The transverse modulus of UB O-WF/PLIMA biocomposites is higher compared to that of neat hot-pressed UB O-WF sheets, although the fiber content of *V*_f_ is lower (Figure S6 and Table 2). The reason is that the PLIMA polymer matrix fills the pores and enhances stress transfer between wood fibers, which is critical for transverse loading.

The random-in-plane WF/PLIMA biocomposites showed higher *E* and σ_U_ values compared to many other wood fiber-based composites, including WF/thermoset composites (Figure 4c). The UB R-WF/PLIMA composite, with 56 wt % (*V*_f_ 51%) WF (Figure 4c), demonstrates the highest σ_U_ and *E* values, at 106 MPa and 11.7 GPa, respectively. In many previous studies in Figure 4c, fiber damage is a problem, whereas the composites presented here have well-preserved fibers. While high fiber content wood fiber/PF (phenol formaldehyde) composites can show superior mechanical properties, such as higher modulus and tensile strength, compared to WF/PLIMA biocomposites,^67^ the toxicity of PF resin due to formaldehyde limits its use. The reinforcement efficiency of the fibers can be discussed based on effective fiber modulus and fiber strength using simple “rule of mixtures” approaches.^68^ This makes it possible to compare different types of materials, even when the fiber content is different. For UB R-WF sheets, the effective fiber modulus (*E*_f_) is estimated at ≈38 GPa. When the PLIMA polymer matrix fills pores and enhances stress distribution, the *E*_f_ is increased from 38 to 52 GPa (Table 2, UB R-WF/PLIMA). Oriented fibers improve absolute mechanical properties, and the presence of a PLIMA matrix increases effective *E*_f_ also for this case. For UB L-WF sheets, *E*_f_ increased from 39 to 55 GPa when the PLIMA matrix is added. The similarity of *E*_f_ obtained for random and oriented fiber distributions is in support of the analysis. The effective fiber strength, σ_f_, can be estimated for the oriented materials. The values are similar for fiber sheets and biocomposites from unbleached fibers and are encouragingly high (σ_f_ = 438 MPa for the network and σ_f_ = 464 MPa for the biocomposite). It means that unbleached fibers result in a strong fiber network. The load is effectively transferred at the microscale in hot-pressed, oriented unbleached fiber networks, and stress is well distributed among individual fibers even without a polymer matrix.

For bleached, oriented fibers, the effective fiber properties are reduced compared to unbleached fibers, especially for the hot-pressed fiber networks. The strong reduction in σ_f_ for B L-WF fiber networks compared to UB L-WF (139 MPa vs 438 MPa) is primarily attributed to insufficient interfiber bonding.

## Conclusions

Optically translucent biocomposites were prepared by liquid molding, where bleached industrial wood fiber networks were impregnated with biobased limonene acrylate LIMA, followed by curing. The permeability was much higher, and impregnation was more convenient than that for delignified wood substrate reinforcements dominating the literature for transparent biocomposites. The eco-indicators (cumulative energy demand and greenhouse gas emissions) were dominated by the biobased acrylic acid used for LIMA. Further reduction in the environmental impact of wood fiber biocomposites should therefore focus on the polymer matrix and the long service life of the product.

Wood fiber networks may be preshaped for molding complex geometrical shapes, a distinct advantage compared to wood veneer biocomposites (“transparent wood”). The optical transmittance was lower than expected (attenuation coefficient of 2.1 cm^–1^ at 35 vol % fiber content), due to the large number of light-scattering PLIMA/wood fiber interfaces from the high content (35 vol %) of collapsed ribbon-like fibers.

Neat, hot-pressed wood fiber networks with low porosity (20–24%) were mechanically robust with *E* = 19 GPa and σ_U_ = 210 MPa at 80 vol % fibers preferentially oriented in the loading direction. Higher lignin content improved the interfiber stress transfer and mechanical properties. Polymer matrix (WF/PLIMA) composites with the same unbleached fiber reinforcement (*E* = 17 GPa, σ_U_ = 140 MPa, *V*_f_ = 47%) showed lower strength due to the lower fiber content. Although the PLIMA resin is brittle, it enhances stress transfer, resulting in an increased effective fiber modulus (*E*_f_ ≈ 55 GPa), and WF/PLIMA composites showed moisture sorption much lower than that of neat fiber networks. For lowered environmental impact, the fiber volume fraction of biocomposites should be increased beyond the 50% achieved in this study.

## Experimental Section

### Wood Fibers and Preparation of Molded Fiber Sheets

Never-dried industrial high-lignin softwood kraft pulp fibers from SCA Munksund AB were used. The fibers’ dimensions were measured by fiber testing equipment (Fibertester Plus, Lorentzen & Wettre, Stockholm, Sweden). The average length of wood fibers was 1.99 mm, and their average width was 34.7 μm.

The kink index was calculated using the number of kinks *N* (*N*_*x*1 – *x*2_ is the number of kinks with an angle between *x*1° and *x*2°) and the total length (*L*_tot_) of all fibers, eq 1. The fibers showed a relatively low kink index of 0.59.

1 $$Kink\;index=\frac{{2N}_{20-50}+{3N}_{50-90}+{4N}_{90-180}}{L_{tot}}$$

The aqueous wood fiber suspension (∼0.3 wt %) was vacuum-filtered into wet sheets using a Finnish sheet former (Lorentzen & Wettre, Stockholm, Sweden) to prepare R-WF or using a dynamic sheet former (Formette Dynamic, Fibertech AB, Sweden) at a drum rotation speed of 1200 rpm to prepare O-WF. The wood fiber sheets were hot-pressed into molded fibers, where the wet fiber sheets were created with random-in-plane fiber orientation (R-WF) or with orientation (O-WF), followed by cold pressing and then hot-pressing (185 °C for 20 min) as described in ref 10, see Figure 1a. Both types of fiber sheets had a basis weight of ∼200 g/m^2^.

Hot-pressed O-WF sheets were bleached according to a similar method reported in ref 71. The bleaching solution was prepared by mixing chemicals in the following order: deionized water, sodium silicate (3.0 wt %), sodium hydroxide solution (3.0 wt %), magnesium sulfate (0.1 wt %), diethylenetriaminepentaacetic acid (0.1 wt %), and then hydrogen peroxide (4.0 wt %). The sheets were immersed in the bleaching solution at 70 °C for 30 min (turned yellowish white) or 2 h (turned white). The samples were then thoroughly washed with water. Note that only the O-WF sheets were bleached since the R-WF sheets disintegrated during bleaching.

### Chemical Composition

Sugar analysis was performed through acid hydrolysis using the Dionex ICS-3000 high-performance anion-exchange chromatography system (Thermo Fisher Scientific Inc., USA), and the Klason lignin content was determined according to the standard TAPPI T222 om-02 from the acid-insoluble fraction. The cellulose and hemicellulose (glucomannan and xylan) contents were calculated based on the detected sugar concentrations.^69^ In the unbleached wood fibers, the content of lignin was 13.7%, while the cellulose, glucomannan, and xylan contents were 67%, 9.7%, and 9.5%, respectively. The wood fiber sheet bleached for 2 h had a lignin content of 8%, while cellulose, glucomannan, and xylan contents were 82.4%, 4.9%, and 4.7%, respectively.

### Synthesis of Limonene Acrylate (LIMA)

LIMA was synthesized via ring-opening acrylation of limonene oxide (1 equiv) with acrylic acid (4 equiv) under solvent-free conditions by letting it react at 75 °C for 3 h. 4-Methoxyphenol was added as an inhibitor (0.04 equiv). After the reaction, the product was cooled down, and the acrylic acid excess was removed by washing it with deionized water and adding Na_2_CO_3_. LIMA was isolated as a transparent oil with a yield of 87% based on limonene oxide.

### Preparation of Transparent Biocomposites from Hot-Pressed Wood Fiber Sheets

The fiber sheets were impregnated with a mixture of LIMA monomer and a radical initiator (2,2′-azobis 2-methylpropionitrile, 0.5 wt %) for 1 h under vacuum. The sheets were soaked in acetone for 10 min prior to impregnation to facilitate wetting and monomer diffusion into the swollen fiber. The LIMA-impregnated sheets were then packaged between two glass slides, wrapped with aluminum foil, and polymerized at 75 °C for 12 h.

Molded fibers were also formed between two cylinders at one capped end. Then, PLIMA impregnation and curing occurred. After the final material is released and the material is trimmed, you can see a more complex shape in Figure 1b, demonstrating the potential of transparent wood fiber biocomposites in creating intricate 3D structures using molded fibers and proper processing techniques.

### Microscopy

A scanning electron microscope (SEM) (TM-1000, Hitachi High-Tech, Japan) was used for morphological studies. The molded fiber sheets and biocomposites were epoxy-embedded and polished to study their cross sections. The samples were sputter-coated for 20–60 s under vacuum with platinum–palladium using a 208HR Cressington Sputter Coater before microscopy.

### Thickness and Tensile Test

Specimens of 6 × 80 mm were cut from the samples and loaded into an Instron 5944 tensile machine (USA), equipped with a 500 N load cell and a video extensometer. The tests were conducted at a 30 mm gauge length and a cross-head speed of 5 mm/min. The samples were conditioned for 48 h at 23 °C and 50% relative humidity prior to the test. At least five specimens were tested for each sample.

### Optical Properties

Optical measurements were conducted by using an integrating sphere in the 450–800 nm wavelength region. A Quartz Tungsten Halogen light source (model 66181 from Oriel Instruments), with a stable output in this region, was used. Visible transmittance and haze were measured by following ASTM D1003.

### Wide-Angle X-ray Diffraction

The X-ray machine used Ni-filtered Cu Kα radiation with a wavelength of 1.542 and a Philips PW3830 generator operating at 30 kV and 20 mA. The beam had a diameter of 300 μm. The X-ray beam was perpendicular or parallel (lengthwise, i.e., parallel to wood fiber orientation or widthwise) to the biocomposite surface. For each sample, the exposure time was set to 1 h. The diffraction patterns were recorded on Fujifilm imaging plates and read with a Fujifilm BAS-1800II bioimaging analyzer. Two-dimensional wide-angle X-ray diffraction (WAXD) patterns were detected. The 2D X-ray patterns were reduced to 1D profiles by C++ programming.

The crystallinity was calculated using the one-dimensional WAXD intensity against 2θ curves. The crystallite size was determined by analyzing the position of crystal peaks (θ = Bragg angle), their full width at half-maximum (FWHM), the X-ray wavelength (λ), and applying the Scherrer equation (eq 2).

2 $$D_{P}=\frac{0.94\lambda }{FWHM\times cos\left(\theta \right)}$$

Hermans’ orientation factor (*f*) of the (2 0 0) crystalline plane was calculated from intensity-azimuthal angle distributions using eqs 2 and 3.^70^ The Debye–Scherrer ring’s intensity, *I*(φ), and the azimuthal angle, φ, were used to determine the value of *f*. A value of *f* = 1 represents the strongest orientation in the longitudinal direction, while a value of *f* = −0.5 signifies the highest orientation of cellulose crystals in the transverse direction.

3 $$f=\frac{3\left\langle \cos^{2}\left(\varphi \right)\right\rangle -1}{2}$$

4 $$\left\langle \cos^{2}\varphi \right\rangle =\frac{\int_{0}^{\pi /2}I\left(\varphi \right)\;\sin\left(\varphi \right){\;\cos}^{2}\left(\varphi \right)\;d\varphi }{\int_{0}^{\pi /2}I\left(\varphi \right)\;\sin\left(\varphi \right)\;d\varphi }$$

## Supplementary Material



## List of Acronyms and Terms with Background Information

Acronyms and terms with background information can be found in Table 3.

**Table 3:** Acronyms, Terms, and Background Information

acronym	full form	role in the manuscript	brief background information
B O-WF/PLIMA	bleached oriented wood fibers/poly(limonene acrylate)	transparent composite with bleached oriented fibers	bleached fibers improve transparency but reduce strength due to weaker interfacial bonding
CED	cumulative energy demand	eco-indicator for material production	quantifies energy use, e.g., 60 MJ/kg for PLIMA, for sustainability assessment
ESBO	epoxidized soybean oil	alternative biobased monomer or prepolymer for polymer synthesis	produced by epoxidizing double bonds in soybean oil; serves as a reactive component in polymer synthesis
epoxy-embedded	epoxy-embedded	sample preparation for microscopy	stabilizes samples for SEM, enabling precise microstructure analysis
FTIR	Fourier transform infrared spectroscopy	chemical analysis of fiber composition	identifies changes like lignin reduction in bleached fibers
GWP	global warming potential	eco-indicator for greenhouse gas emissions	measures emissions to evaluate the environmental impact of biocomposites
in-situ synthesis	in situ synthesis	polymerization within the fiber network	enhances fiber–matrix integration, reduces energy compared to e.g., melt-processing
LIMA	limonene acrylate	biobased monomer for polymer synthesis	from citrus waste, enables eco-friendly polymerization for biobased composites
PHA	polyhydroxyalkanoate	alternative biobased polymer matrix	biopolyesters produced by microorganisms from sugars, fats, or agricultural waste
PLIMA	poly(limonene acrylate)	biobased polymer matrix	transparent thermoset, matches wood’s refractive index, sustainable alternative to PMMA
PMMA	poly(methyl methacrylate)	fossil-based polymer matrix for comparison	an acrylic polymer previously used to prepare TW had a lower refractive index matching with wood fibers compared to PLIMA
SEM	scanning electron microscopy	microstructure imaging	visualizes fiber and matrix distribution, often with epoxy-embedded samples
TW	transparent wood	reference material for comparing optical and mechanical properties	combines wood’s structure with transparency via delignification and polymer infiltration
UB O-WF/PLIMA	unbleached oriented wood fibers/poly(limonene acrylate)	high-strength and modulus composite with unbleached oriented fibers	unbleached oriented fibers with retaining lignin for enhanced mechanical properties, less transparent
WAXD	wide-angle X-ray diffraction	fiber orientation analysis	measures cellulose crystal alignment to assess fiber orientation and dependent properties
WF	wood fibers	reinforcement in composites	high-strength, renewable fibers from pulp mills with low energy demand

## Notes

SEM images and fiber dimensions for unbleached kraft wood fibers and molded fiber sheets (oriented and random-in-plane, surface and cross-section); optical properties: transmittance, haze, and attenuation coefficients for biocomposites; PLIMA film photograph; environmental impact: energy demand and warming potential for unbleached and bleached biocomposites; thermal and mechanical data: DSC curves for PLIMA, transverse tensile curves of biocomposites, and mechanical property comparisons for wood fiber composites (including literature); structural analysis: WAXD patterns, crystallinity indices, crystallite sizes, and orientation parameters for molded fibers and biocomposites; and schematics and calculations: X-ray beam directions, cellulose microfibril orientation, fiber misalignment angles, and effective fiber mechanical properties (PDF)
