# Supplementary material for: Translucent Biocomposites from Hot-Pressed Wood Fibers and Poly(limonene acrylate)
Source: ACS Appl Mater Interfaces. 2025 Jul 21;17(30):43522–35. doi: 10.1021/acsami.5c07130 (PMC12314871; doi:10.1021/acsami.5c07130)
Supplement: Supplementary file 1 [file am5c07130_si_001.pdf]

Supporting Information

Translucent Biocomposites from Hot-Pressed Wood Fibers and Poly(Limonene Acrylate)

Erfan Oliaei[1*], Céline Montanari[1], Lengwan Li[1,2], Hui Chen[1], Peter Olsén[1,3], Lars Berglund[1,3*]

[1]*Wallenberg Wood Science Center, Department of Fibre and Polymer Technology, KTH Royal Institute of Technology, Stockholm 10044, Sweden ([*]oliaei@kth.se; [*]blund@kth.se)*

[2]*State Key Laboratory of Organic-Inorganic Composites, School of Materials Science and Engineering, Beijing University of Chemical Technology, Beijing 100029, China*

[3]*Laboratory of Organic Electronics, Linköping University, Norrköping 60174, Sweden ([*]lars.berglund@liu.se)*

Contents

Fiber Analysis 2

Molded Fibers 4

Optical Properties 4

Environmental Impact Assessment........ 6

Thermal Properties 8

Mechanical Properties 8

Structural Analysis – X-ray 9

Calculation of Effective Fiber Modulus and Strength 15

Moisture Sensitivity 17

Fiber Analysis

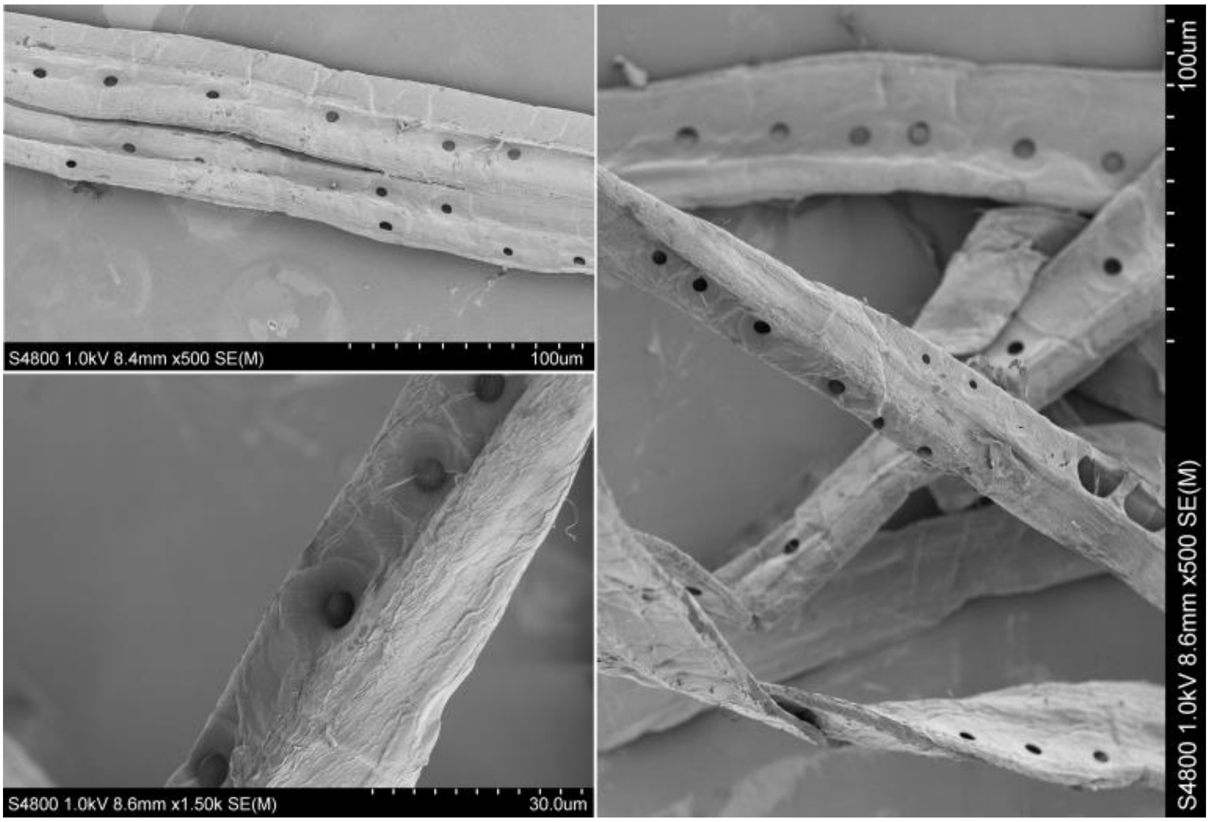

Figure S1. SEM images of unbleached kraft wood fibers.

The fibers were mostly straight with a low kink index of 0.59 (measured by L&W Fibertester Plus). This indicates that they have not been damaged significantly by the industrial process. Cell wall piths are common in softwood fibers and are visible in fibers in this image.

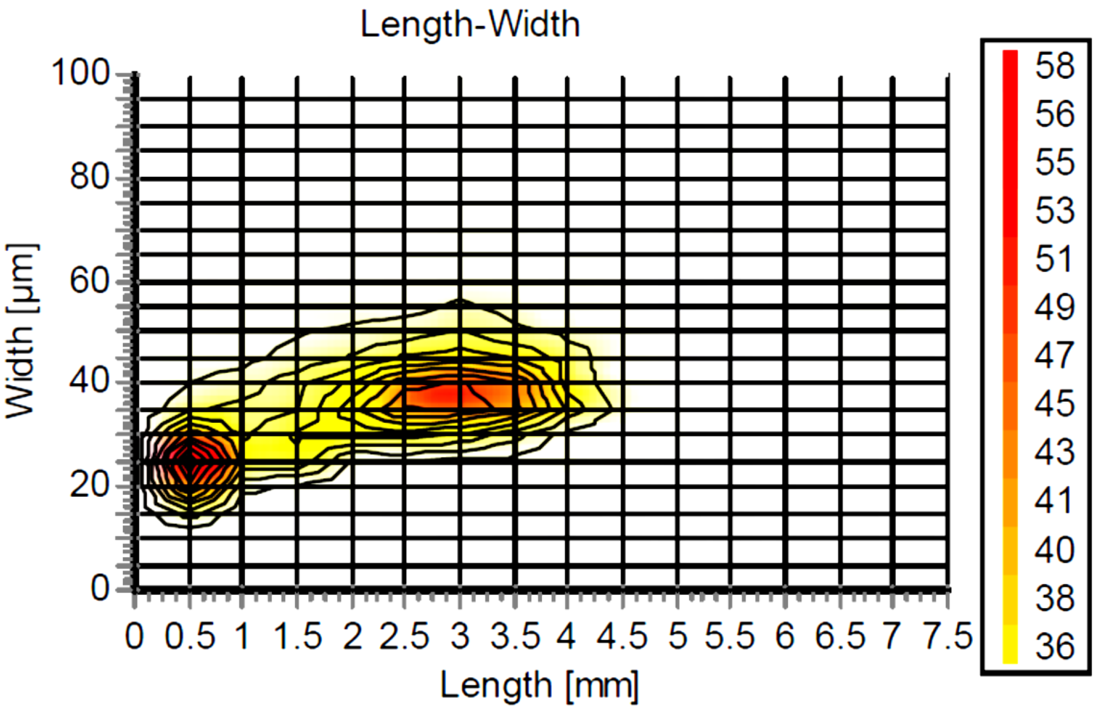

Figure S2. Fiber distribution dimensions were measured in 20,000 counts using an L&W Fibertester Plus. Color codes represent relative fiber per mille.

Molded Fibers

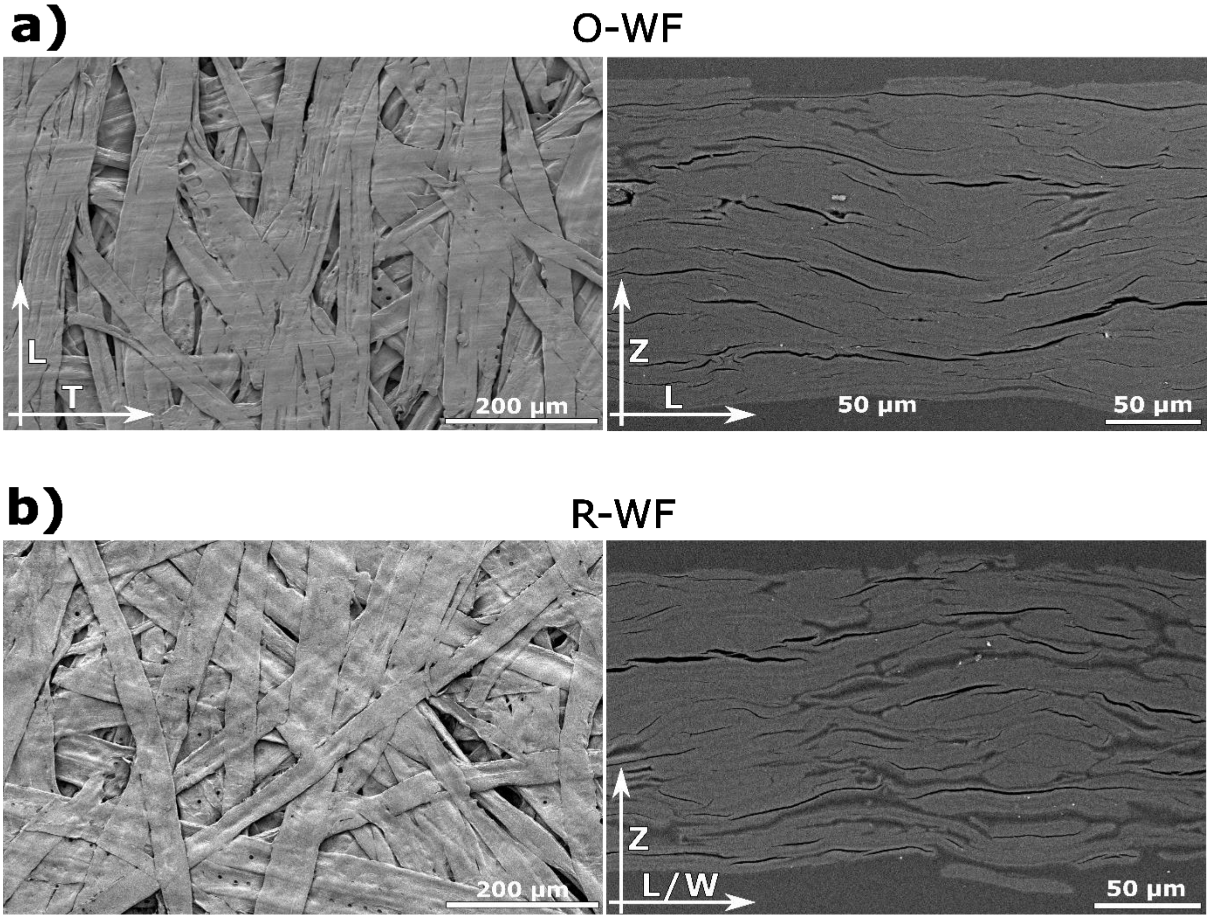

Figure S3. SEM images from the surfaces and cross-sections of a) oriented wood fiber (O-WF) sheets, showing preferred fiber orientation in the machine direction, and b) randomly oriented wood fiber (R-WF) sheets. Some parts of the figure were reconstructed from ref [1].

Optical Properties

Table S1. Optical properties of wood fiber sheets and biocomposites at 550 nm wavelength. See Fig 2b.

Sample	Thickness (µm)	Transmittance (%)	Haze (%)
Unbleached O-WF	~180	2	95
Bleached O-WF (2h)	~190	16	97
Unbleached O-WF biocomposite	~240	22	66
Bleached O-WF biocomposite (30min)	~290	67	60
Bleached O-WF biocomposite (2h)	~300	83	48
PLIMA	~1200	92	2

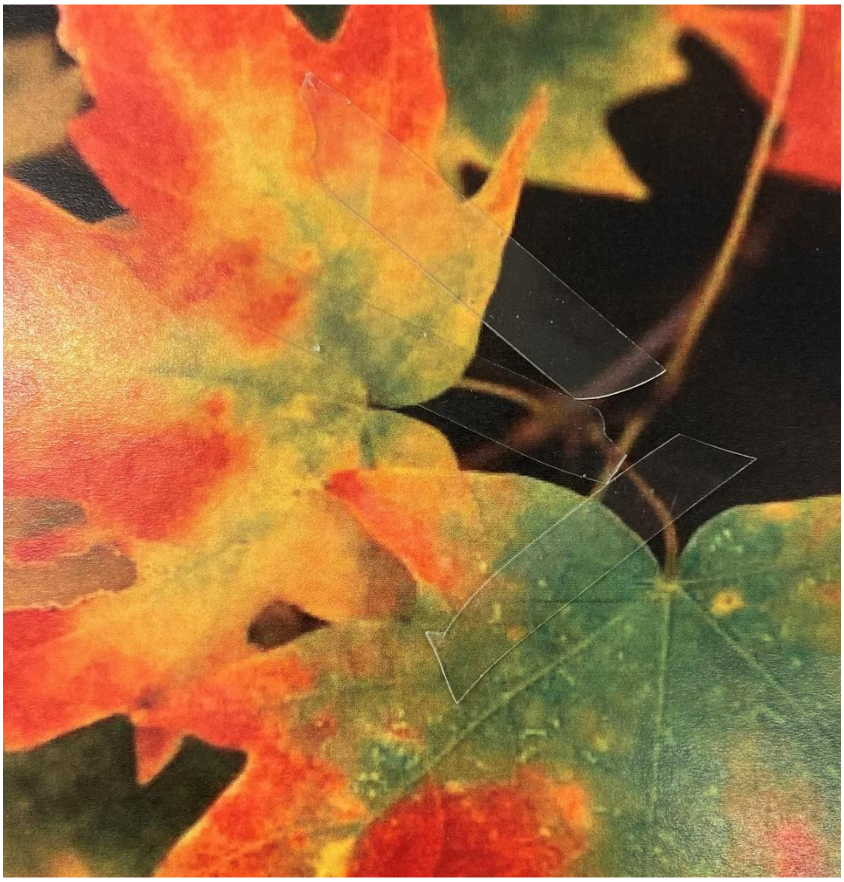

Figure S4. Photograph of several PLIMA film strips with a clear appearance against a background of leaves.

Attenuation coefficient

The total transmittance (T_{total}) of transparent wood (TW) diminishes exponentially with sample thickness, Equation (1).

$$T_{\text{total}} = \exp(-\alpha d) \qquad (1)$$

where α is the attenuation coefficient for wood polymer composites defined as Equation (2)[2]:

$$\alpha = \sqrt{\left(\frac{D_{xy}}{D_z}\right)^{1/6} \frac{\mu}{D_{xy}}} \qquad (2)$$

The attenuation coefficient is reliant not only on the absorption coefficient μ, but also on the anisotropic diffusion coefficients (D_{xy} and D_z; diffusion coefficients in the x-y plane and z-direction). This relationship indicates that the total transmittance decreases exponentially with the sample's thickness d. The attenuation coefficient α is influenced by both the absorption coefficient and the anisotropic diffusion coefficients, highlighting the significance of both absorption and scattering in determining the optical properties of scattering materials such as TW or WF composites. This exponential dependency on thickness is essential for understanding and forecasting the transmittance of these scattering materials.

Although the attenuation coefficient remains unchanged for similar TW materials of different thicknesses, it varies for TW materials with different cellulose contents[2]. Since transmittance is not dependent on thickness, we attempted to roughly calculate the attenuation coefficient for different scattering transparent cellulosic materials, as summarized in Table S2.

To calculate the attenuation coefficient accurately, transmittance must be measured for multiple thicknesses of the same material, using the formula $T_{\text{total}} = \exp(-\alpha d)$, where T_{total} is the total transmittance. α is the attenuation coefficient, and d is the thickness. A linear fit of $\mathrm{Ln}(T_{\text{total}})$ against thickness (d) then allows the determination of α from the slope, confirming the exponential decay relationship. In the current study, however, only two data points are available: the transmittance at a specific thickness and the observation that transmittance is approximately 96% at near-zero thickness. Consequently, the calculation relies only on these two points and should be considered approximate.

Table S2. Attenuation coefficient at 550 nm wavelength for transparent TW or WF scattering materials.

Sample	Reference	α (cm^{-1})
B WF PLIMA (V_f 35%)	**Present work**	2.1
UB WF PLIMA (V_f 51%)	**Present work**	26.6
WF acrylic (V_f ~49%)	3	20.8
WF acrylic (V_f ~23%)	4	7.6
TW PMMA (V_f 12%)	2	1.67
TW PLIMA (V_f 12%)	5	0.4

Environmental Impact Assessment

According to Ref. [6], the energy demand for limonene acrylate (LIMA) preparation combines contributions from limonene oxide, acrylic acid, and direct synthesis. Specifically, 0.745 kg of limonene oxide contributes about 8.45 MJ (0.745 kg × 11.34 MJ/kg), 1.39 kg of acrylic acid supplies roughly 36.19 MJ (1.39 kg × 26.0375 MJ/kg), and the direct energy input for synthesis is 7.02 MJ. For 0.950 kg of LIMA, the total cumulative energy demand (CED) is approximately 51.66 MJ, or about 54.38 MJ/kg LIMA.

In a biobased scenario using acrylic acid (AA) produced from lactic acid in a sugarcane biorefinery, the energy inputs are detailed in recent literature[7]. The process requires around 0.245 MJ/kg AA for heating, 0.190 MJ/kg AA for cooling, and 0.307 kWh/kg AA for electricity—which converts to about 1.105 MJ/kg—totaling 1.54 MJ/kg AA direct energy. Moreover, the biorefinery uses 27.8 t/h of bagasse to produce 10.42 t/h of AA, and with an energy content of about 8 MJ/kg for bagasse, the primary energy input is estimated at 21.3 MJ/kg AA. Thus, the total CED for producing AA is approximately **22.8 MJ/kg**, combining a direct process energy of about 1.5 MJ/kg (excluding upstream biomass cultivation) with a renewable biomass energy input of roughly 21.3 MJ/kg.

Therefore, to produce LIMA from biobased acrylic acid and limonene oxide, 0.745 kg of limonene oxide contributes about 8.45 MJ (0.745 kg × 11.34 MJ/kg), 1.39 kg of acrylic acid supplies roughly 31.69 MJ (1.39 kg × 22.8 MJ/kg), and the direct energy input for synthesis is 7.02 MJ. Summing up these values, the total cumulative energy demand (CED) for producing 0.950 kg of LIMA is approximately 47.16 MJ, or about **49.64 MJ/kg LIMA**.

Table S3. Eco-indicator data for unbleached wood fiber (U-WF) biocomposite.

	**Energy demand (ED) [MJ/kg]**	**Global warming potential (GWP) [kg CO_2 eq./kg]**	**Comments and references**
U-WF	5.2	0.1	[8-11]
Compression molding (Sheet forming & hot-pressing)	5.2	0.3	**ED**: [1, 12-14]<br>**GWP**: ED converted with 226 g.CO_2eq/kWh as means Europe's power grid CO_2 emission[15]
LIMA	49.6	2.2	[6-7]
Resin transfer molding (including vacuum infusion)	2.6	0.2	**ED**: [14]<br>**GWP**: ED converted with 226 g.CO_2eq/kWh as means Europe's power grid CO_2 emission[15]
**Sum**	**62.6**	**2.8**	

ED and GWP are reported per 1 kg finished biocomposite (56 wt% WF, 44wt% PLIMA).

Table S4. Eco-indicator data for bleached wood fiber (B-WF) biocomposite.

	**Energy demand (ED) [MJ/kg]**	**Global warming potential (GWP) [kg CO_2 eq./kg]**	**Comments and references**
U-WF	5.2	0.1	[8-11]
Compression molding (Sheet forming & hot-pressing)	5.2	0.3	**ED**: [1, 12-14]<br>**GWP**: ED converted with 226 g.CO_2eq/kWh as means Europe's power grid CO_2 emission[15]
Bleaching (Chemicals, energy & water)	1.7	0.1	[8-10]
LIMA	49.6	2.2	[6-7]
Resin transfer molding (including vacuum infusion)	2.6	0.2	**ED**: [14]<br>**GWP**: ED converted with 226 g.CO_2eq/kWh as means Europe's power grid CO_2 emission[15]
**Sum**	**64.3**	**2.9**	

ED and GWP are reported per 1 Kg finished biocomposite (56 wt% WF, 44wt% PLIMA).

For laboratory-scale experiments, LIMA impregnation and polymerization require approximately 30 MJ/kg [6], but large-scale resin transfer molding reduces this to 2.6 MJ/kg [14], as shown above in Tables S3 and S4.

Thermal Properties

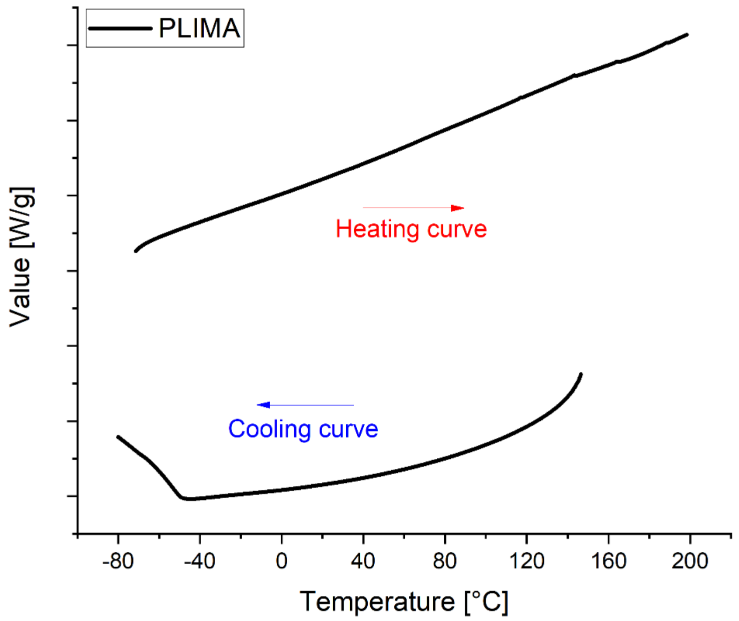

Figure S5. DSC curves of the second heating cycle and cooling cycle of neat PLIMA, show no melting behavior.

Mechanical Properties

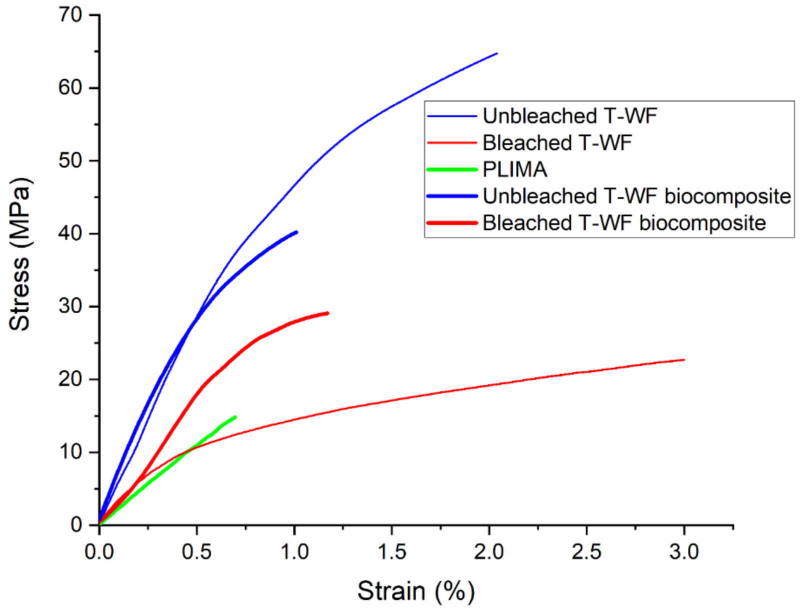

Figure S6. Tensile curves of unbleached and bleached wood fiber sheets and corresponding biocomposites in the transverse direction.

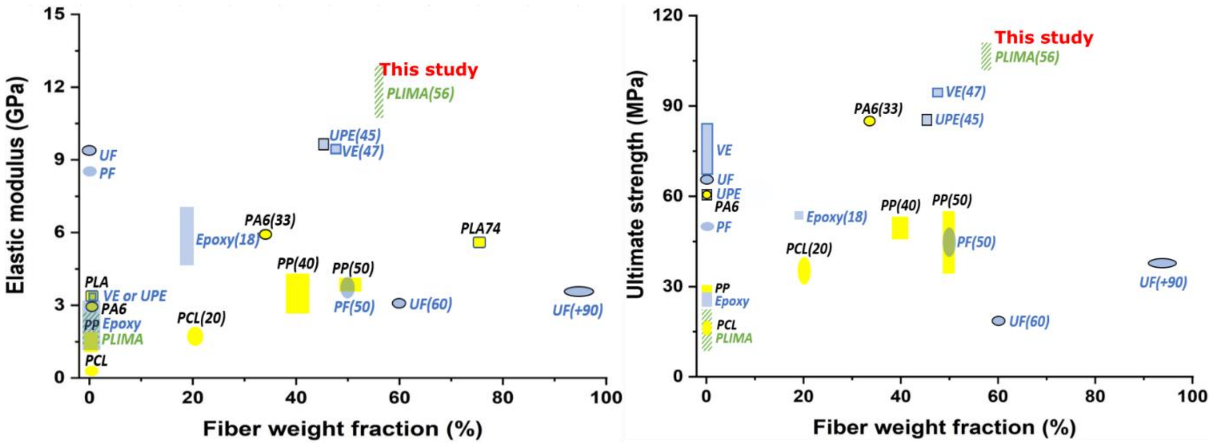

Figure S7. Comparison of elastic modulus and tensile strength of wood fiber (WF) biocomposites in this study to literature WF composites. Random-in-plane fiber reinforcement is used in all composites, with thermoplastic composites in yellow and thermoset composites in blue. Composites are named using the polymer matrix and fiber weight fraction in parentheses. Literature data include vinyl ester (VE)[16-17], urea-formaldehyde (UF)[18-20], unsaturated polyester (UPE)[16, 20], phenol-formaldehyde (PF)[18, 20], epoxy[21-22], polyamide 6 (PA6)[23-24], polylactic acid (PLA)[22], polypropylene (PP)[25-28], and polycaprolactone (PCL)[29] composites. See Fig 3 for a 3D plot.

Structural Analysis – X-ray

The WAXD data revealed the extent of orientation of cellulose crystals (fibrils) in molded wood fibers and PLIMA biocomposites.

Diffraction patterns in Figure S8 (perpendicular beam) show clear anisotropy for O-WF and biocomposites, unlike R-WF samples, which show little anisotropy due to random fiber distribution. For diffraction patterns related to other beam directions refer to Figure S9 and Table S6.

The modest in-plane orientation tendency evident in the R-WF data is because the material comprises randomly distributed fibers. Each fiber possesses a few micrometers thick cell wall with a specific microfibrillar angle orientation, but there are only a few fibers distributed across the thickness of the sheets[1], so the R-WF shows a small tendency of cellulose crystal orientation.

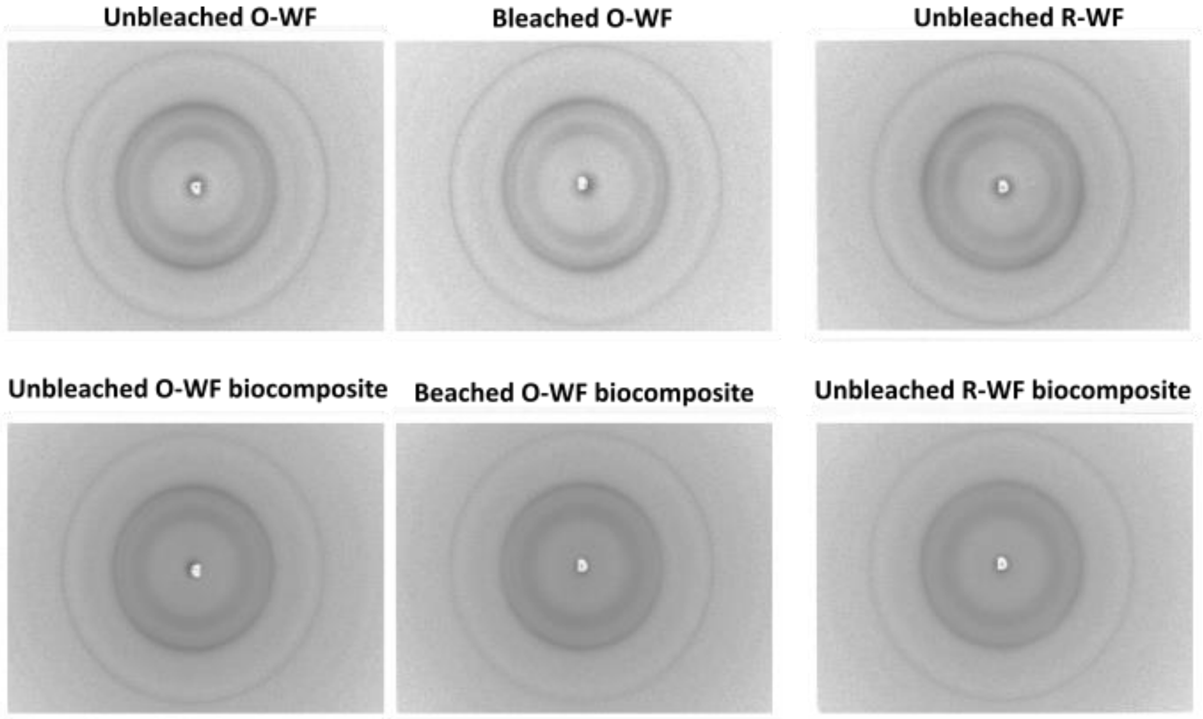

Figure S8. WAXD diffraction patterns of molded wood fibers and PLIMA biocomposites created from random in-plane (R-WF) and oriented (O-WF) fiber sheets (unbleached and bleached), perpendicular to the film. See Figure S9 for other directions.

The analysis conducted in the study focused on the crystallinity, crystallite sizes, and Hermans' orientation parameter (f) of both molded wood fibers and PLIMA biocomposites. The data are summarized in Table S5. Table S5 presents the crystallinity data (related to cellulose) for both molded fibers and PLIMA biocomposites. The degree of crystallinity in unbleached molded fibers is shown to increase after bleaching. This can be attributed to the removal of non-cellulosic amorphous components, such as lignin and hemicellulose. In addition to the increase in the degree of crystallinity, the cellulose crystal size in bleached molded fibers is also observed to increase, due to the agglomeration of cellulose microfibrils, which are more densely bundled[30]. This observation is consistent with previous studies[31-32].

A comparison of the cellulose crystallite sizes between molded fibers and PLIMA biocomposites reveals that the crystallite sizes in the biocomposites are significantly larger. This could be attributed to the in-situ formation of the polymer matrix, which leads to a realignment and agglomeration of cellulose microfibrils in a more densely packed manner. The overall crystallinity in biocomposites is decreased because of the addition of an amorphous polymer matrix. Further discussion on crystal sizes is provided in SI.

The perpendicular to plane Hermans' orientation parameter (f_P) of O-WF was calculated as 0.69-0.70 (Table S5). This value is significantly higher than that of oriented holocellulose molded fibers (0.56)[33], but similar to the f_P of unbleached fibers of O-WF that were previously manufactured (0.71)[1]. The primary factor responsible for the high orientation parameter is the inherent stiffness and straightness of the high-yield unbleached fibers, which align particularly well in the direction of drum rotation.

The cross-sectional Hermans' orientation parameters, lengthwise f_L and widthwise f_W, for O-WF are measured as very high values (f_L = 0.73 and f_W = 0.70). In comparison, R-WF samples (both molded fibers and biocomposites) exhibit a similarly high cross-sectional orientation parameter of

approximately 0.71. As a result, the fraction of fibers exhibiting high out-of-plane orientation or waviness is quite low. This is noteworthy because the in-plane modulus is inversely proportional to the average angle of out-of-plane fiber orientation.

An observation can be made that the orientation parameters for biocomposites are lower than those for molded fibers in any given direction. The main reason for this observation is that the fibers' initial positions have been slightly altered by the in-situ polymerization process of the polymer matrix, leading to a little increased out-of-plane orientation and a slightly more erratic in-plane orientation of the fibers. A similar trend can also be observed with bleached molded fibers, where the orientation parameters are lower compared to unbleached molded fibers. This can also be attributed to the slightly more erratic orientation of the fibers that occurs after the bleaching process.

Table S5. Crystallinity, crystallite sizes, and Hermans' orientation parameter (*f*) of molded wood fibers and PLIMA biocomposites made from random (R-WF) and oriented (O-WF) fiber sheets, both bleached and unbleached. X-ray diffraction measurements were taken from various beam directions: perpendicular (P), lengthwise (L), and widthwise (W) directions to the film. See Table S6 as well.

Sample		Crystallinity index (%)	Crystallite size (nm)		Hermans' orientation parameter (*f*)
			2 0 0	0 0 4	2 0 0
Unbleached O-WF	L	20	4.39	7.29	0.726
	P	18	4.58	8.7	0.693
	W	25	4.29	4.83	0.699
Unbleached O-WF biocomposite	L	13	5.47	9.89	0.717
	P	11	5.43	10.74	0.684
	W	17	4.89	3.48	0.689
Bleached O-WF	L	22	4.5	9.67	0.731
	P	18	4.55	9.57	0.700
	W	29	4.19	3.96	0.701
Bleached O-WF biocomposite	L	12	5.77	8.43	0.708
	P	11	6.32	10.61	0.679
	W	16	4.7	5.08	0.685
Unbleached R-WF	L/W	21	4.58	5.48	0.711
	P	18	4.23	7.77	-
Unbleached R-WF biocomposite	L/W	15	4.93	6.47	0.697
	P	12	5.43	7.64	-

For the random-in-plane samples L and W directions are similar. f_P is not calculated for random-in-plane samples due to the little anisotropy.

The (200) crystal plane is parallel, and the (004) crystal plane is perpendicular to the fibril axis. Consequently, when analyzing each sample using X-ray beam, the apparent (200) crystal size is nearly identical across different angles. In contrast, more (004) crystal planes are detected by the X-ray beam in the lengthwise direction, resulting in a larger apparent crystal size. On the contrary, the apparent (004) crystal size is smaller in the widthwise direction.

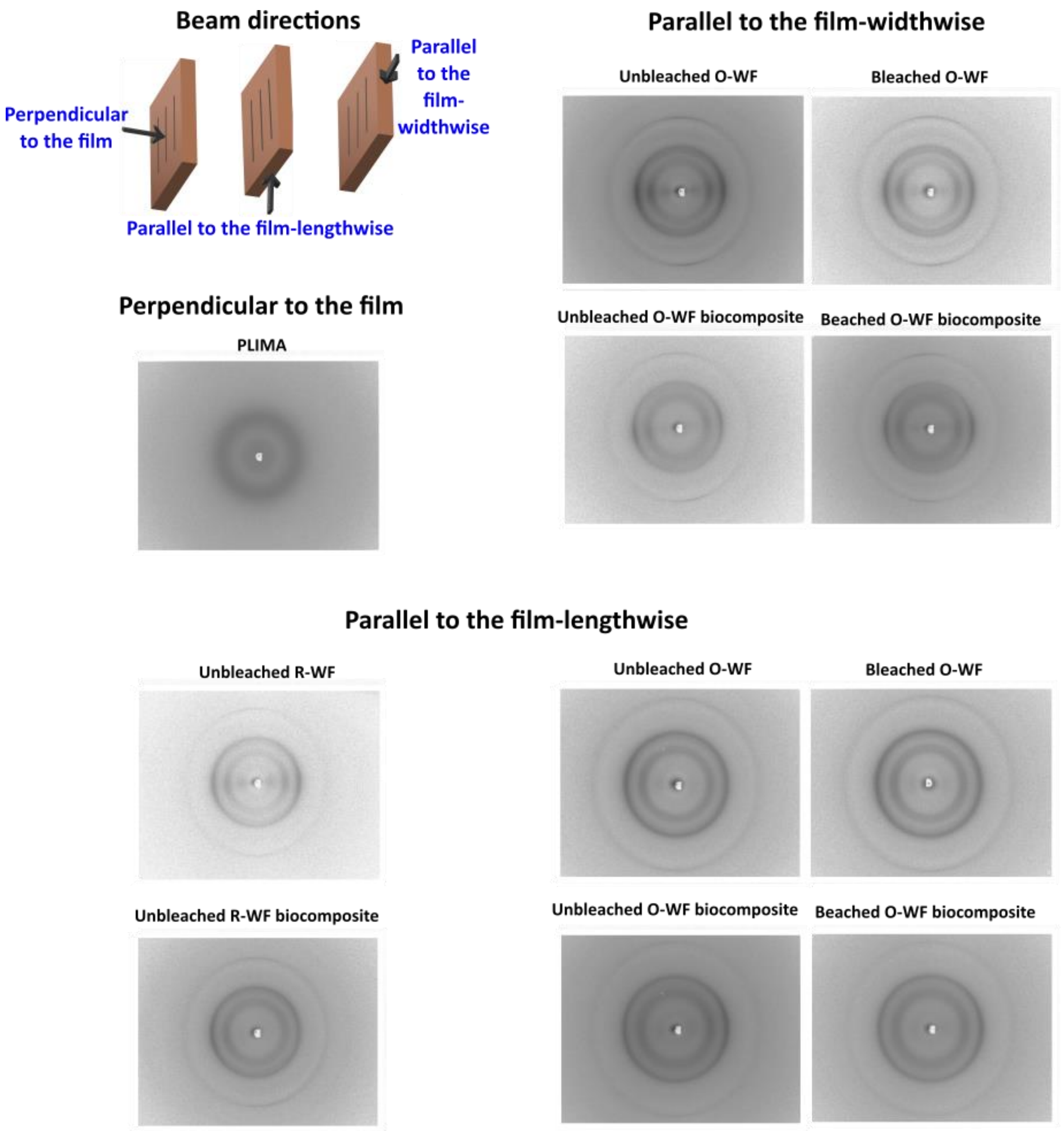

Figure S9. A schematic representation of the beam directions and WAXD diffraction patterns. WAXD diffraction patterns of molded wood fibers and PLIMA biocomposites created from random in-plane (R-WF) and oriented (O-WF) fiber sheets (unbleached and bleached). The patterns shown are from beams perpendicular to the film (refer to Figure S8 for all samples), parallel to the films- widthwise and lengthwise.

Table S6. Hermans' orientation parameter (f) of molded wood fibers and PLIMA biocomposites. X-ray diffraction measurements were taken from lengthwise, widthwise, and perpendicular beam directions.

	Samples	f (110/$\bar{1}$10)	f (200)
Lengthwise	Bleached O-WF	0.7194	0.7313
	Unbleached O-WF biocomposite	0.6935	0.7173

	Unbleached O-WF	0.7177	0.7257
	Bleached O-WF biocomposite	0.6931	0.7076
	PLIMA	-	-
	Unbleached R-WF biocomposite	0.6909	0.6973
	Unbleached R-WF	0.7118	0.7112
Widthwise	Bleached O-WF	0.6971	0.7006
	Unbleached O-WF biocomposite	0.6857	0.6888
	Unbleached O-WF	0.6981	0.6992
	Bleached O-WF biocomposite	0.6844	0.6849
Perpendicular	Bleached O-WF	0.6969	0.6997
	Unbleached O-WF biocomposite	0.6802	0.6843
	Unbleached O-WF	0.6892	0.6934
	Bleached O-WF biocomposite	0.6771	0.6793
	PLIMA	-	-
	Unbleached R-WF biocomposite	-	-
	Unbleached R-WF	-	-

The value of the Hermans orientation factor (*f*) was determined using the intensity, I(ϕ), and azimuthal angle, ϕ, of the Debye-Scherrer ring obtained from WAXS experiments. The formula used to calculate f is shown in Equation (3):

$$f = \frac{3\cos^2\varphi - 1}{2} \qquad (3)$$

For unbleached O-WF, f = 0.6934 (200, perpendicular), which corresponds to an average crystal angle of ⟨ϕ⟩ = 26.9°.

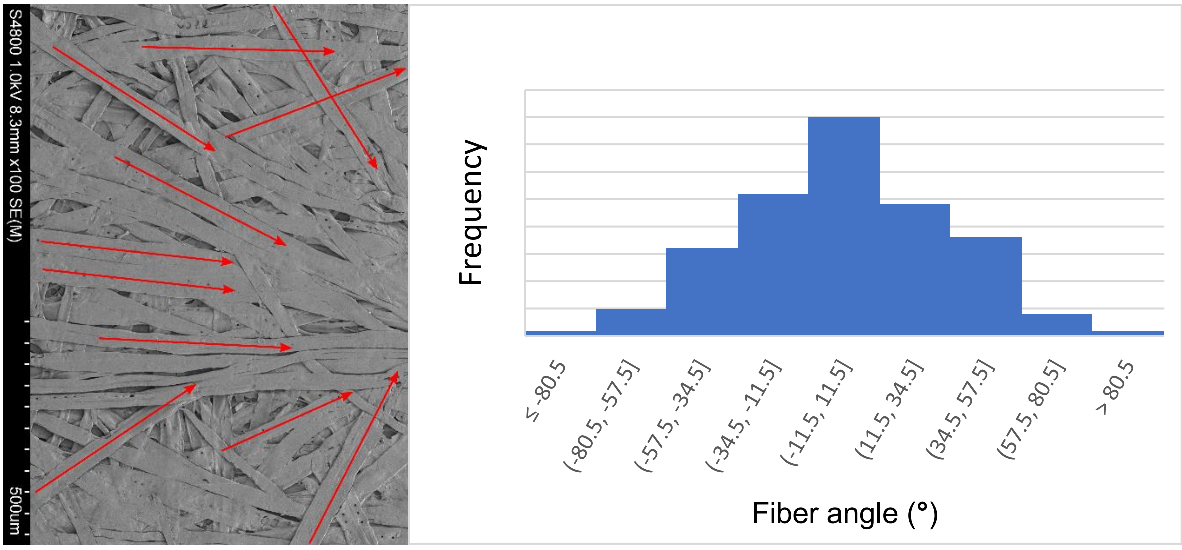

Figure S10. SEM image and fiber misalignment angle distribution for unbleached O-WF (average misalignment to fiber orientation ~26.07°).

The weight average misalignment angle of fibers from the preferred orientation angle was calculated as follows: the average was determined by summing the product of the frequency times mid-misalignment angle of each range (column), divided by the sum of the frequencies. Two average misalignment angles were calculated for this purpose: one for positive angles only, and another for negative angles only.

$$\frac{\left(1\times\frac{103.5+80.5}{2}\right)+\left(4\times\frac{80.5+57.5}{2}\right)+\left(18\times\frac{57.5+34.5}{2}\right)+\left(24\times\frac{34.5+11.5}{2}\right)+\left(\frac{40}{2}\times\frac{11.5-11.5}{2}\right)}{1+4+18+24+\frac{40}{2}} = 26.09°$$

$$\frac{\left(1\times\frac{-103.5-80.5}{2}\right)+\left(5\times\frac{-80.5-57.5}{2}\right)+\left(16\times\frac{-57.5-34.5}{2}\right)+\left(26\times\frac{-34.5-11.5}{2}\right)+\left(\frac{40}{2}\times\frac{-11.5+11.5}{2}\right)}{1+5+16+26+\frac{40}{2}} = -26.04°$$

The resulting weight average misalignment angle of fibers was found to be 26.07°, which agrees well with the average crystal misalignment angle calculated from the Hermans orientation factor, <ϕ>=26.9°. This is due to the helical organization of microfibrils around the fiber axis. Each fiber contributes a positive average microfibril angle (+MFA) related to the top cell wall layer and a negative average microfibril angle (-MFA) related to the bottom cell wall layer, and the overall contribution aligns with the fiber axis (see Figure S11).

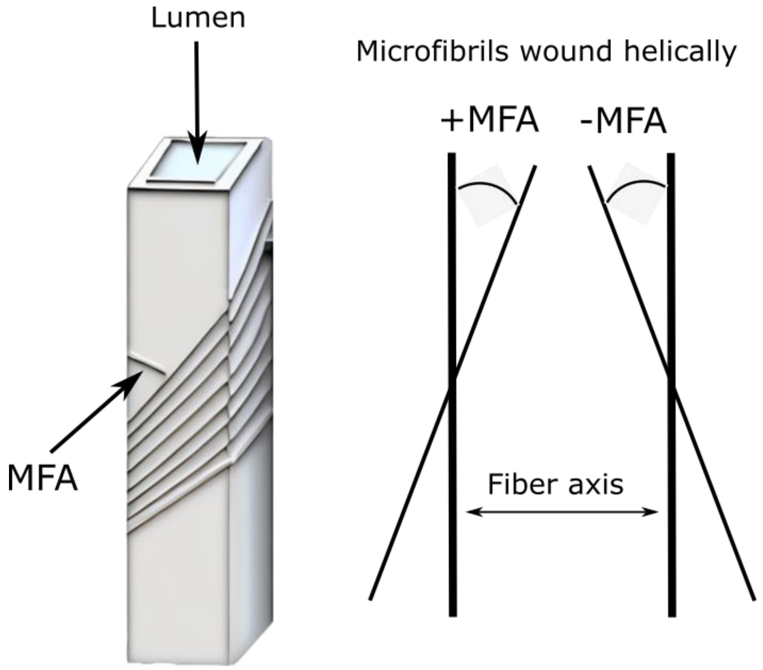

Figure S11. Schematic representation of a single wood fiber and the orientation of the cellulose microfibrils relative to the fiber axis, indicated on both front (+MFA) and back (-MFA) sides of the fiber. Image reconstructed from [34].

Calculation of Effective Fiber Modulus and Strength

Rule of mixtures is the simplest micromechanical model used to predict the composite elastic modulus parallel to the principal axis, based on the assumption that fibers and matrix will experience equal strain under loading in the fiber direction. The modulus of a continuous unidirectional fiber composite in the fiber direction is given by Equation (4)[35]

$$\mathrm{E}_{composite,L} = \mathrm{E}_{matrix} \times \nu_{mtrix} + \mathrm{E}_{fiber} \times \nu_{fiber} \qquad (4)$$

If the fibers are not unidirectional, a correction for orientation is needed.

$$\mathrm{E}_{composite,L} = \mathrm{E}_{matrix} \times \nu_{mtrix} + \eta_0 \mathrm{E}_{fiber} \times \nu_{fiber} \qquad (5)$$

If we have an orientation distribution, we can use formula below for correction according to Krenchel[36] to account for different fiber orientations, Equation (6).

$$\eta_0 = \sum\left(\nu_{f\,i} \cdot cos^4\theta_i\right) \qquad (6)$$

Regarding effective fiber strength in longitudinal direction ($\sigma_{f,L}$), we used a similarly modified rule of mixtures (similar to Cox-Krenchel model for modulus).

Note that for the transverse direction, we have not calculated effective properties due to the limitations of the assumptions and because fiber fracture is not typically observed in that mode.

The orientation efficiency factor in the longitudinal direction ($\eta_{0,L}$) is calculated as:

$$\eta_{0,L} = \frac{1}{69}\cos^4\left(\frac{103.5+80.5}{2}\right) + \frac{4}{69}cos^4\left(\frac{80.5+57.5}{2}\right) + \frac{18}{69}cos^4\left(\frac{57.5+34.5}{2}\right) + \frac{24}{69}cos^4\left(\frac{34.5+11.5}{2}\right) + \frac{20}{69}cos^4\left(\frac{11.5-11.5}{2}\right)$$

which approximates to

$\eta_{0,L} \approx 2.14 \times 10^{-8} + 0.000956 + 0.0609 + 0.249 + 0.290 \approx 0.60$

According to Kaw (2005)[37], the modulus of a random in-plane fiber composite can be expressed as Equation (7):

$$\mathrm{E}_{\text{random}} = \frac{3}{8}\mathrm{E}_{11} + \frac{5}{8}\mathrm{E}_{22} \qquad (7)$$

where:

- E11 is the longitudinal modulus of a unidirectional fiber composite, modeled using the rule of mixtures, Equation (4): $\mathrm{E}_{11} = \mathrm{E}_{fiber} \times \nu_{fiber} + \mathrm{E}_{matrix} \times \nu_{matrix}$
- E22 is the transverse modulus of the unidirectional composite, modeled using the inverse rule of mixtures: $\mathrm{E}_{22} = \frac{\mathrm{E}_{fiber,\mathrm{T}} \times \mathrm{E}_{matrix}}{\mathrm{E}_{fiber,\mathrm{T}} \times \nu_{matrix} + \mathrm{E}_{matrix} \times \nu_{fiber}}$, with $\mathrm{E}_{fiber,\mathrm{T}}$ representing the transverse modulus of the wood fiber.

Therefore,

$$\mathrm{E}_{\text{random}} = \frac{3}{8}\left(\mathrm{E}_{fiber} \times \nu_{fiber} + \mathrm{E}_{matrix} \times \nu_{matrix}\right) + \frac{5}{8}\left(\frac{\mathrm{E}_{fiber,\mathrm{T}} \times \mathrm{E}_{matrix}}{\mathrm{E}_{fiber,\mathrm{T}} \times \nu_{matrix} + \mathrm{E}_{matrix} \times \nu_{fiber}}\right) \qquad (8)$$

Experimental studies[38-39] report that $\mathrm{E}_{fiber,\mathrm{T}}$ ≈ 2 GPa for wood fibers.

Table S7. Mechanical and physical properties of the WF sheets and biocomposites.

Samples	V_f (%)*	Modulus (GPa)	Ultimate strength (MPa)	Effective fiber modulus $E_{f,L}$ (GPa)	Effective fiber strength $\sigma_{f,L}$ (MPa)
PLIMA	0	2.2 (0.4)	15 (7)	-	-
UB R-WF	76	10.8 (0.4)	118 (7)	37.9	-
UB R-WF/PLIMA	51	11.7 (1.1)	106 (5)	52.2	-
UB L-WF	80	18.7 (0.5)	210 (6)	39.0	437.5
B L-WF	76	13.0 (1.5)	67 (5)	28.5	139.0
UB L-WF/PLIMA	47	16.7 (2.5)	139 (9)	55.1	464.4
B L-WF/PLIMA	35	11.6 (1.3)	77 (5)	48.4	320.2
UB T-WF	80	6.0 (0.6)	65 (4)	-	-
B T-WF	76	3.0 (0.3)	23 (1)	-	-
UB T-WF/PLIMA	47	6.7 (1.0)	41 (2)	-	-
B T-WF/PLIMA	35	4.1 (0.1)	28.2 (1.2)	-	-

Moisture Sensitivity

A controlled humidity chamber with 99% relative humidity (RH) at 23 °C was created using a saturated solution of potassium sulfate (K_2SO_4) in water. Test specimens were conditioned in a sealed plastic chamber containing the potassium sulfate solution for up to 16 days. Baseline condition was 50% RH and 23 °C. Wood fiber sheets, composites, and PLIMA samples were evaluated for moisture sensitivity, specifically dimensional swelling (thickness change measured at a minimum of 12 points using a micrometer and averaged) and moisture absorption (weight gain measured with a precision balance). Measurements were recorded at various time intervals up to 16 days. At least three specimens per sample were tested.

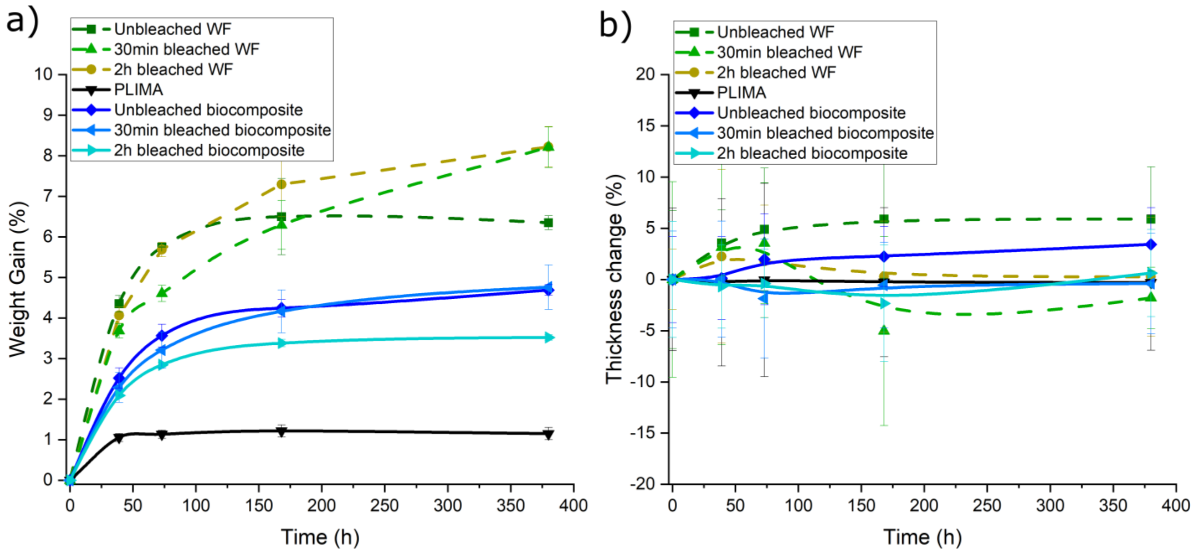

Figure S12. Moisture absorption and swelling effects after conditioning at 99% RH (23 °C; baseline: 50% RH) for wood fiber sheets, composites, and PLIMA. a) Moisture absorption shown as normalized weight gain over conditioning time. b) Swelling shown as normalized thickness change over conditioning time.

Wood fiber biocomposites incorporating PLIMA show significantly lower moisture uptake compared to pure wood fiber sheets, with weight gains typically around 3–4% versus 6–8% after 16 days under 99% RH at 23°C (Figure S12a). This reduction is primarily due to the hydrophobic polymer matrix, which offers interfacial protection and limits water penetration into the fiber network. No significant difference in moisture absorption was observed between unbleached and bleached wood fiber sheets, although the bleached samples showed a slightly higher increase (8% compared to 6%). PLIMA showed exceptional resistance to moisture, with minimal water uptake of approximately 1.2% weight gain over 16 days.

In terms of dimensional stability, changes in thickness were generally small, with a tendency for biocomposites to exhibit slightly less swelling/change than pure wood fiber sheets. Although the measurements showed considerable variability, this was largely due to local thickness variation within each sample rather than experimental error. This trend, indicated by solid versus dashed curves in thickness measurements (Figure S12b), again highlights the role of matrix protection in reducing fiber swelling. The wood fiber sheets showed the greatest tendency for thickness change (~5%), whereas neat PLIMA showed the most stable performance with minimal thickness change.

References

1. Jungstedt, E.; Oliaei, E.; Li, L.; Östlund, S.; Berglund, L. A., Mechanical behavior of all-lignocellulose composites—Comparing micro- and nanoscale fibers using strain field data and FEM updating. *Composites Part A: Applied Science and Manufacturing* **2022,** *161*, 107095. DOI: 10.1016/j.compositesa.2022.107095.
2. Chen, H.; Baitenov, A.; Li, Y.; Vasileva, E.; Popov, S.; Sychugov, I.; Yan, M.; Berglund, L., Thickness dependence of optical transmittance of transparent wood: chemical modification effects. *ACS Applied Materials & Interfaces* **2019,** *11* (38), 35451-35457. DOI: 10.1021/acsami.9b11816.
3. Zha, L.; Yan, M.; Berglund, L. A.; Zhou, Q., Tailoring the Holocellulose Fiber/Acrylic Resin Composite Interface with Hydrophobic Carboxymethyl Cellulose to Enhance Optical and Mechanical Properties. *Biomacromolecules* **2024,** *25* (6), 3731-3740. DOI: 10.1021/acs.biomac.4c00295.
4. Yano, H.; Sasaki, S.; Shams, M. I.; Abe, K.; Date, T., Wood Pulp-Based Optically Transparent Film: A Paradigm from Nanofibers to Nanostructured Fibers. *Advanced Optical Materials* **2014,** *2* (3), 231-234. DOI: https://doi.org/10.1002/adom.201300444.
5. Montanari, C.; Ogawa, Y.; Olsén, P.; Berglund, L. A., High Performance, Fully Bio-Based, and Optically Transparent Wood Biocomposites. *Advanced Science* **2021,** *8* (12), 2100559. DOI: https://doi.org/10.1002/advs.202100559.
6. Montanari, C.; Chen, H.; Lidfeldt, M.; Gunnarsson, J.; Olsén, P.; Berglund, L. A., Sustainable thermal energy batteries from fully bio-based transparent wood. *Small 19*, 2301262. DOI: https://doi.org/10.1002/smll.202301262.
7. Brobbey, M. S.; Louw, J.; Görgens, J. F., Biobased acrylic acid production in a sugarcane biorefinery: A techno-economic assessment using lactic acid, 3-hydroxypropionic acid and glycerol as intermediates. *Chemical Engineering Research and Design* **2023,** *193*, 367-382. DOI: https://doi.org/10.1016/j.cherd.2023.03.034.
8. Oliaei, E.; Berthold, F.; Berglund, L. A.; Lindström, T., Eco-friendly high-strength composites based on hot-pressed lignocellulose microfibrils or fibers. *ACS Sustainable Chemistry & Engineering* **2021,** *9* (4), 1899-1910. DOI: 10.1021/acssuschemeng.0c08498.
9. Oliaei, E.; Lindström, T.; Berglund, L. A., Sustainable development of hot-pressed all-lignocellulose composites—comparing wood fibers and nanofibers. *Polymers* **2021,** *13* (16), 2747. DOI: 10.3390/polym13162747.
10. Brundage, A.; Evans, C.; Lizas, D.; Freed, R. *OECD global forum on environment: sustainable materials management*; Organisation for Economic Co-operation and Development (OECD). Mechelen, Belgium, 2010; https://www.oecd.org/env/waste/49804908.pdf (accessed 2020/10/01).
11. Suhr, M.; Klein, G.; Kourti, I.; Rodrigo Gonzalo, M.; Giner Santonja, G.; Roudier, S.; Delgado Sancho, L. *Best available techniques (BAT) reference document for the production of pulp, paper and board*; ISBN 978-92-79-48167-3 (PDF), ISSN 1831-9424 (online); Publications Office of the European Union: Luxembourg, 2015; DOI: 10.2791/370629; https://eippcb.jrc.ec.europa.eu/sites/default/files/2019-11/PP_revised_BREF_2015.pdf (accessed 2025/04/05).
12. Ashby, M. F., Appendix B Eco- and supply-chain. In *Materials and the Environment, Eco-Informed Material Choice*, Elsevier: 2021.
13. Hill, C.; Norton, A., LCA database of environmental impacts to inform material selection process. JCH Industrial Ecology Ltd: 2018.
14. Energy, U. S. D. o. *Bandwidth Study on Energy Use and Potential Energy Savings Opportunities in U.S. Glass Fiber Reinforced Polymer Manufacturing*; U.S. Department of Energy, Advanced Manufacturing Office: Washington, D.C., 2017; https://www.energy.gov/sites/prod/files/2019/05/f62/GFRP_bandwidth_study_2017.pdf (accessed.
15. Christian Redl, F. H., Matthias Buck, Dr. Patrick Graichen, Dave Jones *The European Power Sector in 2020*; 2021. https://ember-climate.org/app/uploads/2022/01/European-Power-Sector-in-2020.pdf (accessed May 2023).

16. Du, Y.; Wu, T.; Yan, N.; Kortschot, M. T.; Farnood, R., Pulp fiber-reinforced thermoset polymer composites: Effects of the pulp fibers and polymer. *Composites Part B: Engineering* **2013,** *48*, 10-17. DOI: https://doi.org/10.1016/j.compositesb.2012.12.003.
17. Friberg, E.; Olsson, J. *Application of Fibre Reinforced Polymer Materials in Road Bridges—General Requirements and Design Considerations*. Master's thesis, Chalmers University of Technology, Gothenburg, Sweden, 2014.
18. Orji, B. O.; McDonald, A. G., Flow, curing and mechanical properties of thermoset resins – wood-fiber blends for potential additive-manufacturing applications. *Wood Material Science & Engineering* **2023**, 1-18. DOI: 10.1080/17480272.2022.2155873.
19. Antov, P.; Savov, V.; Trichkov, N.; Krišťák, Ľ.; Réh, R.; Papadopoulos, A. N.; Taghiyari, H. R.; Pizzi, A.; Kunecová, D.; Pachikova, M., Properties of High-Density Fiberboard Bonded with Urea–Formaldehyde Resin and Ammonium Lignosulfonate as a Bio-Based Additive. *Polymers* **2021,** *13* (16), 2775.
20. Kopeliovich, D. *SubsTech Dokuwiki*. http://www.substech.com/dokuwiki (accessed May 9, 2025).
21. Ansari, F.; Sjöstedt, A.; Larsson, P. T.; Berglund, L. A.; Wågberg, L., Hierarchical wood cellulose fiber/epoxy biocomposites – Materials design of fiber porosity and nanostructure. *Composites Part A: Applied Science and Manufacturing* **2015,** *74* (Supplement C), 60-68. DOI: https://doi.org/10.1016/j.compositesa.2015.03.024.
22. Almgren, K. M. Wood-fibre composites: Stress transfer and hygroexpansion. KTH Royal Institute of Technology, Stockholm, Sweden, 2010.
23. Sears, K. D.; Jacobson, R. E.; Caulfield, D. F.; Underwood, J. Methods of making composites containing cellulosic pulp fibers. U.S. Patent 6,730,249, 2004.
24. Rowell, R. M., *Handbook of Wood Chemistry and Wood Composites*. CRC Press, 2005.
25. Rosenstock Völtz, L.; Di Guiseppe, I.; Geng, S.; Oksman, K., The Effect of Recycling on Wood-Fiber Thermoplastic Composites. *Polymers* **2020,** *12* (8), 1750.
26. Vilaseca, F.; Méndez, J. A.; López, J. P.; Vallejos, M. E.; Barberà, L.; Pèlach, M. A.; Turon, X.; Mutjé, P., Recovered and recycled Kraft fibers as reinforcement of PP composites. *Chemical Engineering Journal* **2008,** *138* (1), 586-595. DOI: https://doi.org/10.1016/j.cej.2007.07.066.
27. Méndez, J. A.; Vilaseca, F.; Pèlach, M. A.; López, J. P.; Barberà, L.; Turon, X.; Gironès, J.; Mutjé, P., Evaluation of the reinforcing effect of ground wood pulp in the preparation of polypropylene-based composites coupled with maleic anhydride grafted polypropylene. *Journal of Applied Polymer Science* **2007,** *105* (6), 3588-3596. DOI: https://doi.org/10.1002/app.26426.
28. Stark, N. M.; Rowlands, R. E., Effects of wood fiber characteristics on mechanical properties of wood/polypropylene composites. *Wood and fiber science* **2003,** *35* (2), 167-174.
29. Lo Re, G.; Sessini, V., Wet Feeding Approach for Cellulosic Materials/PCL Biocomposites. In *Biomass Extrusion and Reaction Technologies: Principles to Practices and Future Potential*, American Chemical Society: 2018; pp 209-226.
30. Newman, R. H., Carbon-13 NMR evidence for cocrystallization of cellulose as a mechanism for hornification of bleached kraft pulp. *Cellulose* **2004,** *11* (1), 45-52. DOI: 10.1023/B:CELL.0000014768.28924.0c.
31. Phan, H. N. Q.; Leu, J. H.; Tran, K. T.; Nguyen, V. N. D.; Nguyen, T. T., Rapid fabrication of pineapple leaf fibers from discarded leaves by using electrolysis of brine. *Textiles* **2023,** *3* (1), 1-10.
32. Daicho, K.; Saito, T.; Fujisawa, S.; Isogai, A., The crystallinity of nanocellulose: dispersion-induced disordering of the grain boundary in biologically structured cellulose. *ACS Applied Nano Materials* **2018,** *1* (10), 5774-5785. DOI: 10.1021/acsanm.8b01438.
33. Yang, X.; Berthold, F.; Berglund, L. A., High-density molded cellulose fibers and transparent biocomposites based on oriented holocellulose. *ACS Applied Materials & Interfaces* **2019,** *11* (10), 10310-10319. DOI: 10.1021/acsami.8b22134.
34. Tabet, T. A.; Aziz, F. A., Cellulose microfibril angle in wood and its dynamic mechanical significance. In *Cellulose-fundamental aspects* **2013**, 113-142.

35. Kollár, L. P.; Springer, G. S., *Mechanics of Composite Structures*. Cambridge University Press: Cambridge, 2003.
36. Krenchel, H., *Fibre reinforcement : theoretical and practical investigations of the elasticity and strength of fibre-reinforced materials*. Alademisk Forlag, 1964.
37. Kaw, A. K., *Mechanics of composite materials*. CRC Press, 2005.
38. Bergander, A.; Salmén, L., The transverse elastic modulus of the native wood fibre wall. *Journal of Pulp and Paper Science* **2000,** *26* (6), 234.
39. Czibula, C.; Brandberg, A.; Cordill, M. J.; Matković, A.; Glushko, O.; Czibula, C.; Kulachenko, A.; Teichert, C.; Hirn, U., Estimation of the in-situ elastic constants of wood pulp fibers in freely dried paper via AFM experiments. *arXiv preprint arXiv:2012.03037* **2020**.
